# Quantitative trait loci for energy balance traits in an advanced intercross line derived from mice divergently selected for heat loss

**DOI:** 10.7717/peerj.392

**Published:** 2014-05-27

**Authors:** Larry J. Leamy, Kari Elo, Merlyn K. Nielsen, Stephanie R. Thorn, William Valdar, Daniel Pomp

**Affiliations:** 1 Department of Biological Sciences, University of North Carolina at Charlotte, Charlotte, NC, USA; 2 Department of Animal Science, University of Nebraska, Lincoln, NE, USA; 3 Department of Genetics, University of North Carolina, Chapel Hill, NC, USA

**Keywords:** QTL by sex interactions, Metabolic rate, Feed intake, Body weight and body composition

## Abstract

Obesity in human populations, currently a serious health concern, is considered to be the consequence of an energy imbalance in which more energy in calories is consumed than is expended. We used interval mapping techniques to investigate the genetic basis of a number of energy balance traits in an F_11_ advanced intercross population of mice created from an original intercross of lines selected for increased and decreased heat loss. We uncovered a total of 137 quantitative trait loci (QTLs) for these traits at 41 unique sites on 18 of the 20 chromosomes in the mouse genome, with X-linked QTLs being most prevalent. Two QTLs were found for the selection target of heat loss, one on distal chromosome 1 and another on proximal chromosome 2. The number of QTLs affecting the various traits generally was consistent with previous estimates of heritabilities in the same population, with the most found for two bone mineral traits and the least for feed intake and several body composition traits. QTLs were generally additive in their effects, and some, especially those affecting the body weight traits, were sex-specific. Pleiotropy was extensive within trait groups (body weights, adiposity and organ weight traits, bone traits) and especially between body composition traits adjusted and not adjusted for body weight at sacrifice. Nine QTLs were found for one or more of the adiposity traits, five of which appeared to be unique. The confidence intervals among all QTLs averaged 13.3 Mb, much smaller than usually observed in an F_2_ cross, and in some cases this allowed us to make reasonable inferences about candidate genes underlying these QTLs. This study combined QTL mapping with genetic parameter analysis in a large segregating population, and has advanced our understanding of the genetic architecture of complex traits related to obesity.

## Introduction

Energy balance in biological organisms is achieved when the amount of energy consumed equals that expended. While energy consumption consists simply of the number of calories eaten, energy is expended both internally in the production of heat and externally during physical exercise (Schoeller, 2009). The maintenance of an appropriate energy balance clearly is critical since increased weight gain leading to obesity can occur if more energy is consumed than expended.

Much of our knowledge of the genetics of obesity has come from discovery of many quantitative trait loci (QTLs) located throughout the genome in mice that affect traits such as body weight, weight gain, and especially various measures of fat (Cheverud et al., 2001; Rocha et al., 2004; Leamy, Pomp & Lightfoot, 2009b; Leamy et al., 2012; Kelly et al., 2011; Cheverud et al., 2011). While fewer studies in mice have been conducted for energy consumption and expenditure, the basic components of energy balance, several QTLs have been found for these traits as well. For food intake measures, it is interesting that many of the QTLs found thus far map to different sites than those affecting body weight and adiposity measures (Allan, Eisen & Pomp, 2005; Kelly et al., 2010; Leamy et al., 2012). This also appears to be the case for QTLs affecting energy expenditure as assessed from voluntary exercise (primarily wheelrunning) traits in mice (Lightfoot et al., 2007; Lightfoot et al., 2008; Lightfoot et al., 2010; Leamy, Pomp & Lightfoot, 2008; Leamy, Pomp & Lightfoot, 2009a; Leamy, Pomp & Lightfoot, 2009b; Nehrenberg et al., 2010; Kelly et al., 2010; Mathes et al., 2011).

Measures of energy expenditure related to metabolic rate have rarely been subjected to QTL analyses in rodent models. A notable exception is heat loss measured by indirect calorimetry. This trait was analyzed by Moody et al. (1999) who made use of two F_2_ mouse populations derived from lines which had undergone divergent selection for high and low heat loss. Mice from the high line had increased heat loss but also tended to be more active with less body fat than mice from the low line (Nielsen et al., 1997b), suggesting a genetic link of heat loss with body fat. And in fact Moody et al. (1999) discovered five significant and four suggestive QTLs for heat loss, several of which mapped in the confidence intervals of QTLs found for different measures of fat in these mice, especially the percentage of brown adipose tissue.

Leamy et al. (2005) estimated genetic parameters for heat loss, food intake, and body weight and composition traits in an F_11_ advanced intercross population (AIL; Darvasi & Soller, 1995) derived from crosses of mice from inbred versions of the high and low heat loss selection lines used by Moody et al. (1999). Heritability estimates for these traits varied, but suggested that a reasonable amount of genetic variability had been preserved in the development of this population from the selection lines. There also were some interesting patterns among the genetic correlations; for example, heat loss was positively associated with food intake but negatively associated with adiposity (Leamy et al., 2005). This population therefore appeared to be an ideal one for a comprehensive QTL study aimed at identifying genes for adiposity and associated energy balance traits. We report here the results of such a study conducted to search for QTLs affecting all of these traits, and to discover their patterns of effects. We were particularly interested in differentiating QTLs acting on these traits independently of overall body size, and therefore analyzed body composition traits both adjusted and not adjusted for body weight at sacrifice.

## Materials and Methods

### The population and traits

We used an advanced intercross (AIL-F11) population of mice originally developed from lines selected for low (ML) and high (MH) heat loss during a 16 generation period (Nielsen et al., 1997a; Nielsen et al., 1997b) of rearing in a vivarium maintained at 23.5 °C. This selection was successful in achieving a divergence of ∼50% in heat loss, 20.6% for feed intake per unit metabolic size, and 40% for body fat percentage (Nielsen et al., 1997a; Nielsen et al., 1997b). Mice were randomly sampled from each of the two selection lines and full-sib matings were done for seven generations to establish mostly inbred high (MHI) and low heat loss (MLI) lines. An intercross of these two inbred lines then was made and continued for 8 generations at which time the population was divided into two replicates and at generation 10 into four replicates. Single-pair matings in generation 10 were replicated, producing F_11_ mice in 8 different groups (4 replicates each with 2 parities). Litter sizes were standardized to 8 at birth, and all pups were weaned at 3 weeks of age. All procedures involving the rearing and husbandry of the mice were approved by the Institutional Animal Care and Use Committee at the University of Nebraska—Lincoln (Protocol 02-02-008).

Altogether, a total of 18 traits were measured: 8 whole body traits (body weights at 4 different ages, 2 weight gain traits, heat loss and feed intake), and 10 body composition traits (4 measures of fat, 3 organ weights, and 3 bone traits). We also collected brains from all mice for possible functional analyses, but with all the other data collected at dissection, we did not have the human resources and time available to weigh the brains. Table 1 gives a list of the 18 traits and their abbreviations and more detailed descriptions of the measurements may be found in Leamy et al. (2005). Although our primary interest was in the energy balance (heat loss and feed intake) and adiposity traits, they often are associated with body weight and composition (organ weight and bone) traits that we therefore examined as well. For example, heat loss is a proxy for basal metabolic rate which in several studies has been shown to be correlated with measures of bone such as bone mineral density (BMD).

**Table 1:** Basic statistics for the traits used in the QTL analysis. Shown are the sample size (*N*), mean, and standard deviation (Std Dev) for each of the 24 traits (and their units and abbreviations) measured in the F_11_ mice.

Trait (units)	Abbreviation	*N*	Mean	Std Dev
3-week body weight (g)	WT3	1513	14.41	1.940
6-week body weight (g)	WT6	1518	28.71	2.539
12-week body weight (g)	WT12	1511	33.61	2.984
Weight gain from 3 to 6 weeks (g)	GAIN3-6	1506	14.31	1.866
Weight gain from 6 to 12 weeks (g)	GAIN6-12	1504	4.87	1.863
Feed intake (g/kg^0.75^/day)	INTAKE	1504	84.23	1.717
Heat Loss (kcal/kg^0.75^/day)	HL	1525	146.77	15.836
Total body fat (g)	FAT	1520	4.37	0.838
Subcutaneous fat pad (g)	SUBQ	1523	0.127	0.038
Gonadal fat pad (g)	GON	1523	0.157	0.074
Brown adipose tissue (g)	BAT	1517	0.045	0.012
Liver weight (g)	LIVER	1520	1.69	0.223
Heart weight (g)	HEART	1519	0.187	0.032
Spleen weight (g)	SPLEEN	1519	0.112	0.028
Total body fat as % of kill weight	PFAT	1520	13.52	2.169
Subcutaneous fat pad as % of kill weight	PSUBQ	1523	0.394	0.111
Gonadal fat pad as % of kill weight	PGON	1523	0.472	0.208
Brown adipose tissue as % of kill weight	PBAT	1517	0.140	0.036
Liver weight as % of kill weight	PLIVER	1520	5.24	0.463
Heart weight as % of kill weight	PHEART	1519	0.581	0.097
Spleen weight as % of kill weight	PSPLEEN	1519	0.350	0.078
Bone mineral density (g/cm^2^)	BMD	1456	0.062	0.003
Bone mineral content (g)	BMC	1456	0.735	0.054
Bone area (cm^2^)	BAREA	1456	11.78	0.667

### Genotyping and molecular markers

SNPs were selected from the Wellcome-CTC Mouse Strain SNP Genotype Set (http://mus.well.ox.ac.uk/mouse/INBREDS). DNA samples from five MHI and five MLI mice representing a subset of the parents used to make this AIL were included in the original Wellcome-CTC genotyping of 13370 SNPs, and from these data we selected 768 evenly spaced SNPs that were predicted to be fully informative within the AIL population based on fixed alternative genotypes between these MHI and MLI mice. These SNPs were genotyped using Illumina Goldengate technology by the Illumina FastTrack service lab (San Diego, CA). Of the 768 SNPs designed for the array, 658 provided data representing high quality and full informativity between the MHI and MLI parental lines.

Appendix S1 provides a listing of all 658 markers with their positions (in Mb) on each of the 20 chromosomes. The mapping resolution in the F_11_ population was enhanced because of a 4.4-fold expansion of the genome. The frequencies of the three genotypes at each of the SNPs on each chromosome are illustrated in Appendix S2. This figure shows that heterozygote frequencies across most chromosomes consistently track around the expected frequency of 50%, as do both homozygotes around their expected frequency of 25%, with some variability as expected.

### Preliminary analyses

We created 10 additional traits by adjusting each of the 10 body composition traits for body weight at sacrifice (WTFINAL). For the 7 non-bone traits, this was accomplished by dividing each value by WTFINAL and then multiplying by 100 to express these values as percentages (traits were designated PFAT, PLIVER, etc.). This was particularly useful in allowing us to directly compare our QTL results for these traits to those from other mouse QTL studies that also used percentages (Cheverud et al., 2001; Gordon et al., 2008; Kelly et al., 2011; Leamy et al., 2012). We adjusted each of the bone traits (designated BMD^a^, BMC^a^, and BAREA^a^) by using WTFINAL as a covariate in the QTL analysis (see below). This allowed us to compare our QTL results for BMD^a^ to those found by Leamy et al. (2013) who also adjusted BMD in their mouse population in this same fashion. Beyond these comparisons with other studies, use of the adjusted body composition traits allowed us to discover QTLs affecting these traits that were independent of overall body weight.

Prior to the QTL analyses, we tested each of the 28 total traits for potential effects of several variables. Multivariate analyses of variance showed significant effects of sex, replication, and parity for all the traits, as did the 20 body composition traits for cohort and the age at sacrifice as well. For food intake (INTAKE) and heat loss (HL), body weight at the time the mouse entered the calorimeter also was significant. Additional significant covariates for HL included the percentage of body weight lost in the calorimeter and the amount of food (g) remaining in the calorimeter, and a random factor, the calorimeter unit in which each mouse was placed. After appropriate adjustment by these covariates and factors, we calculated basic statistics (means and standard deviations) for each of these traits (Table 1). Because of some original technical problems with the PIXImus, occasional mortality among the mice, a few measurement difficulties and recording errors, as well as the number of mice available for genotyping, total sample sizes varied from 1456 to 1525 among the traits.

### QTL mapping

We used the QTLRel program implemented in R (Cheng et al., 2010; Cheng et al., 2011) to map QTLs for each of the 28 traits. The QTL program accounted for the structural relatedness among individuals in our advanced intercross population by calculating identity coefficients (Lynch & Walsh, 1998) from the pedigree information supplied. We used information only from generations 7 to 11 since the relative contribution of earlier generations to the overall amount of inbreeding achieved was considered marginal. As in our previous studies (Leamy et al., 2012; Leamy et al., 2013), we used the Haley–Knott interval mapping (Haley & Knott, 1992) option in QTLRel to impute genotypic values between SNPs separated by more than 1 cM. At all actual and imputed markers, QTLRel evaluated the phenotypic values of each trait with a model that included additive and dominance genetic effects as well as all appropriate covariates (sex, replication, etc.) and factors outlined above to adjust for their potential effects. For all markers on each of the 20 chromosomes, the program calculated likelihood ratio values that were converted into LOD (likelihood of odds) scores.

We evaluated all of the LOD scores generated for each trait by estimating both 5% (significant) and 10% (suggestive) experimentwise thresholds in QTLRel with the permutation method of Churchill & Doerge (1994). Genotypic (rather than phenotypic) values were shuffled in QTLRel program so that the family structures were maintained. We ran this permutation procedure with 1000 iterations and recorded the 95th and 90th percentile LOD values in each of these runs. The 95th percentile values were used as the 5% experimentwise (significant) thresholds and the 90th percentile values were used as the suggestive threshold values.

Beyond these permutations used to correct for the many tests performed at each marker throughout the genome, it also was necessary to adjust for the QTL scans run for the 28 traits. To accomplish this, we first recorded the lowest probability from the scans for each of the 28 traits. These 28 probabilities then were subjected to the false discovery rate (FDR) procedure, assuming dependence among the traits (Benjamini & Hochberg, 1995). This procedure indicated that the probability of false QTLs was less than 0.05 for all LOD scores 4.8 or higher, less than 10% for all LOD scores greater than 4.45 and less than 20% for LOD scores greater than 4.1.

We considered the highest LOD score on each chromosome that reached the suggestive threshold value as representing the site of a putative QTL. Multiple LOD score peaks exceeding this value on the same chromosome also were regarded as potential QTL sites if the peaks were separated by a drop of at least 1.5 LOD units. We also used QTLRel to estimate confidence intervals for each of the QTLs that were defined by 1.5 LOD drops on either side of the peak position (Manichaikul et al., 2006).

At the site of each putative QTL, QTLRel computed additive (*a*) and dominance genotypic values (*d*) and tested these values for significance (*P* < 0.05). These values were computed from probabilities so were subject to possible inflation. The additive genotypic value estimates one-half of the difference between the phenotypic values for the two homozygotes and thus is useful in describing the magnitude of effect of each QTL. The dominance genotypic values estimate the difference between the mid-homozygous and the heterozygous values, and where significant, suggest that those QTLs exhibit dominance (Falconer & Mackay, 1996). If *d* values approximately equal *a* values, this suggests complete dominance whereas *d* values greater than + *a* values (or less than −*a* values) indicate overdominance (Falconer & Mackay, 1996). QTLRel also estimated the percentage of the total phenotypic variation of the trait explained by each QTL.

Once the locations of all putative QTLs were determined, we used QTLRel to test for their potential interactions with sex. This was done by the calculation of a probability associated with the difference between likelihood values produced in models run with and without a sex by QTL interaction. Any of these probabilities less than the conventional 0.05 level were considered to be statistically significant (Kenney-Hunt et al., 2008; Leamy et al., 2012). Where these interactions occurred, we tested the effect of the QTL in the separate sexes and used the suggestive threshold value to assess significance.

## Results

Results of the QTL analyses are presented in Tables 2–4. LOD scores for half (68) of the 137 total QTLs identified exceeded 4.8, suggesting that the probability of any of these being false is less than 5%. The probability of false positives is less than 10% for 81 QTLs with LOD scores greater than 4.45 and 20% for 106 QTLs with LOD scores greater than 4.1. In general, therefore, the probability is high that the majority of the QTLs found are genuine and do not represent false positive results.

**Table 2:** QTL results for the whole body traits measured in the **F_11_** mice. Shown are all QTLs affecting the traits measured on the live F_11_ mice that had LOD scores reaching the 10% (suggestive) or 5% (†) experimentwise level of significance. Locations on each chromosome (Ch) and confidence intervals of the QTLs are given in Mb (from NCBI Build 37). Also shown is the percentage contribution (%) of each QTL to the total variance of each trait, and its additive (*a*), dominance (*d*) genotypic effects (bolded values indicate significance at *P* < 0.05). Interactions of QTLs with sex are indicated as M (significant in males only), F (significant in females only) or both M and F (significant in both sexes where bolded values indicate the sex for which the QTL had the greater effect).

Trait	Ch	Location	Conf. Interval	LOD	*a*	*d*	%	Sex
HL	1	128.7	111.0–133.5	6.87^†^	**3.8402**	−0.2316	2.50	
	2	25.6	12.8–29.4	5.09^†^	**3.4861**	−0.3666	1.95	
INTAKE	5	108.0	106.2–111.8	4.55^†^	**−2.6615**	−0.7727	1.71	
WT3	8	77.9	68.5–85.8	3.81	**0.2604**	**0.2858**	0.89	**M**, F
	12	80.8	78.1–82.9	4.88^†^	**0.3361**	−0.0772	1.47	
	13	57.4	54.7–60.5	4.03	**0.3366**	0.0138	1.25	
	X	50.4	48.3–54.8	4.57^†^	**−0.2413**	0.0828	1.11	
	X	78.8	68.8–88.9	6.43^†^	**−0.3716**	−0.0631	2.41	
	X	101.4	100.0–103.0	19.49^†^	**−0.6090**	0.0961	5.77	
WT6	2	104.5	98.0–108.7	4.54^†^	**0.4045**	0.226	0.73	
	6	125.7	116.8–128.9	4.01	**0.4737**	0.0835	0.85	**M**, F
	7	124.3	116.8–132.2	4.13^†^	**−0.6451**	−0.5557	1.93	
	12	80.8	78.1–82.9	10.19^†^	**0.7008**	−0.2101	1.92	**M**, F
	13	57.4	54.7–59.5	5.21^†^	**0.5295**	0.1007	1.03	
	X	66.3	58.1–67.2	9.93^†^	**−0.6627**	−0.2258	2.38	M
	X	107.7	107.7–124.7	35.24^†^	**−1.1317**	**0.4093**	5.37	**M**, F
WT12	2	101.4	101.2–114.5	3.86	**0.4607**	−0.2314	0.62	
	2	147.5	146.5–152.2	4.05	**0.5261**	−0.2354	0.97	M,**F**
	5	139.8	134.4–142.3	4.02	**0.4095**	**0.8124**	1.06	
	8	77.9	66.6–83.3	4.76^†^	**0.6204**	0.2274	0.79	
	12	77.0	74.8–81.9	6.19^†^	**0.6702**	−0.0032	1.10	M
	13	57.4	56.6–65.0	5.77^†^	**0.6759**	0.1217	1.10	
	X	66.3	58.1–68.5	8.67^†^	**−0.7503**	−0.312	2.06	M
	X	118.9	104.2–125.8	20.43^†^	**−1.1371**	0.1121	3.57	**M**, F
WTFINAL	2	21.4	5.8–25.6	4.05	**0.6495**	0.0971	1.08	M,**F**
	7	139.1	136.4–141.9	4.88^†^	**−0.6036**	−0.1701	1.09	
	8	82.5	67.8–86.8	3.83	**0.4983**	−0.0121	0.65	**M**, F
	12	80.8	77.1–82.9	8.92^†^	**0.7630**	−0.1584	1.59	M
	13	58.9	41.0–65.0	4.04	**0.5537**	0.0895	0.79	
	17	43.1	29.7–50.7	4.19^†^	**0.5164**	−0.0162	0.74	
	X	66.3	58.1–67.2	11.67^†^	**−0.8473**	0.1347	2.50	M
	X	118.9	108.6–125.8	22.60^†^	**−1.1271**	0.2942	3.66	**M**, F
GAIN3-6	6	118.4	113.5–123.4	4.96^†^	**0.3491**	**0.1819**	0.83	**M**, F
	7	124.5	118.9–132.6	4.40^†^	**−0.5311**	−0.3709	1.94	
	12	76.9	71.3–86.9	4.14^†^	**0.3508**	−0.105	0.77	M
	X	107.7	107.7–126.4	12.59^†^	**−0.5524**	0.2551	2.02	M
GAIN6-12	3	31.4	27.2–41.1	3.88	**0.3248**	0.1302	1.30	M

**Table 3:** QTL results for the body composition traits measured in the F_11_ mice. Shown are all QTLs affecting the body composition traits measured in the F_11_ mice that had LOD scores reaching the 10% (suggestive) or 5% (^†^) experimentwise level of significance. Locations on each chromosome (Ch) and confidence intervals of the QTLs are given in Mb (from NCBI Build 37). Also shown is the percentage contribution (%) of each QTL to the total variance of each trait, and its additive (a), dominance (d) genotypic effects (bolded values indicate significance at *P* < 0.05). Interactions of QTLs with sex are indicated as M (significant in males only), F (significant in females only) or both M and F (significant in both sexes where bolded values indicate the sex for which the QTL had the greater effect).

Trait	Ch	Location	Conf. Interval	LOD	*a*	*d*	%	Sex
FAT	2	19.7	11.6–26.2	5.45^†^	**0.2148**	0.0548	2.49	F
	3	99.8	83.1–114.7	4.64^†^	**−0.1248**	**0.2227**	2.83	
	6	124.0	118.8–126.4	5.44^†^	**0.1691**	−0.0325	1.75	
	10	75.3	65.0–91.5	4.18^†^	**−0.1435**	−0.0349	1.24	
	17	48.6	34.9–57.1	4.49^†^	**0.2135**	0.0262	2.45	
	X	54.2	48.3–58.1	5.45^†^	**−0.1337**	0.0583	1.49	
PFAT	1	25.5	21.4–35.8	3.75	**−0.3262**	0.0436	1.13	
	2	21.0	12.8–29.4	3.98	**0.4411**	0.1667	1.93	
	3	95.6	80.6–102.1	8.98^†^	**−0.5424**	**0.5023**	5.22	
	6	124.0	117.4–126.6	4.50^†^	**0.3774**	−0.0858	1.61	
	10	72.7	67.4–92.7	4.42^†^	**−0.4117**	−0.0143	1.76	
	X	107.7	101.5–126.4	4.45^†^	**0.3491**	−0.1171	1.35	
SUBQ	2	19.4	12.7–29.0	7.42^†^	**0.0120**	0.0017	4.78	
	3	81.6	79.7–106.9	4.40^†^	**−0.0062**	0.0030	1.72	
	6	124.0	119.0–126.6	4.27^†^	**0.0070**	−0.0019	1.91	
	10	92.0	82.7–100.0	4.56^†^	**−0.0091**	**−0.0091**	4.32	
	17	48.6	34.9–62.7	3.77	**0.0093**	0.0014	2.88	
PSUBQ	2	19.4	11.5–29.4	5.92^†^	**0.0314**	0.0067	3.56	
	3	81.6	79.7–100.9	6.57^†^	**−0.0229**	0.0091	2.39	
	10	92.0	84.0–98.2	4.76^†^	**−0.0270**	**−0.0271**	4.17	
	10	127.2	114.0–130.3	3.81	**−0.0227**	−0.0035	2.16	
GON	6	124.0	118.0–126.3	6.48^†^	**0.0175**	−0.0011	2.02	M
	X	58.4	54.1–66.5	4.01	**−0.0265**	−0.0002	1.06	M
PGON	3	95.6	79.5–104.5	4.46^†^	**−0.0280**	**0.0627**	3.01	
	6	124.0	117.4–126.3	6.11^†^	**0.0477**	0.0090	2.18	**M**, F
	X	109.9	101.5–126.4	6.38^†^	**0.0467**	−0.0147	2.10	
BAT	6	125.7	122.6–128.9	4.13^†^	**0.0024**	0.0001	1.77	
PBAT	X	107.7	104.1–125.8	3.85	**0.0056**	0.0010	1.52	
LIVER	2	74.1	69.2–78.9	4.40^†^	**0.0443**	−0.0045	1.32	F
	2	104.5	98.0–106.7	4.05^†^	**0.0344**	**0.0222**	0.95	
	2	140.1	135.9–146.2	4.47^†^	**0.0458**	−0.0077	1.74	
	7	122.7	114.4–129.8	4.88^†^	**−0.0689**	−0.0335	3.14	
	8	83.6	81.6–85.8	3.80	**0.0390**	−0.0110	1.06	
	17	44.2	31.6–49.8	3.85	**0.0410**	0.0080	1.11	
PLIV	1	96.7	89.4–117.1	3.82	**0.0663**	**0.1151**	2.79	
	6	99.5	91.7–112.4	4.58^†^	**−0.1243**	**−0.0939**	3.33	
	11	102.1	98.8–107.8	4.55^†^	**−0.0889**	−0.0333	2.46	
	19	7.5	5.3–10.2	4.01	**0.0760**	−0.0205	1.53	M, **F**
	X	107.7	108.6–125.8	6.70^†^	**0.0963**	0.0292	2.79	
HEART	3	77.1	70.0–77.1	4.08^†^	**0.0041**	**0.0056**	0.99	
	8	127.6	126.4–130.1	4.09^†^	**0.0065**	−0.0009	1.67	
SPLEEN	1	127.2	124.4–133.5	4.11^†^	**0.0047**	−0.0026	1.76	
	8	74.8	51.2–80.8	6.91^†^	**0.0072**	0.0002	2.88	
	12	81.9	78.3–82.9	19.26^†^	**0.0098**	**−0.0087**	7.82	
	17	38.0	31.8–49.4	10.47^†^	**0.0085**	−0.0017	4.79	
PSPLEEN	1	127.2	123.3–131.6	4.57^†^	**0.0149**	−0.0056	1.63	
	8	67.4	58.5–82.5	5.40^†^	**0.0170**	−0.0018	1.74	M, **F**
	12	81.9	80.8–84.7	16.23^†^	**0.0234**	**−0.0254**	5.03	
	17	38.0	31.8–49.4	8.49^†^	**0.0207**	−0.0046	2.84	M, **F**
	19	4.0	4.0–6.5	3.81	**0.0090**	**−0.0114**	1.10	M, **F**
	X	124.7	106.8–126.4	9.50^†^	**0.0185**	**0.0169**	3.46	M, **F**
	X	136.6	133.1–137.8	9.41^†^	**0.0167**	**0.0177**	3.11	M, **F**

**Table 4:** QTL results for the bone traits measured in the F_11_ mice. Shown are all QTLs affecting the unadjusted and adjusted (*a*) bone traits measured on the F_11_ mice that had LOD scores reaching the 10% (suggestive) or 5% (^†^) experimentwise level of significance. Locations on each chromosome (Ch) and confidence intervals of the QTLs are given in Mb (from NCBI Build 37). Also shown is the percentage contribution (%) of each QTL to the total variance of each trait, and its additive (*a*), dominance (*d*) genotypic effects (bolded values indicate significance at *P* < 0.05). Interactions of QTLs with sex are indicated as M (significant in males only), F (significant in females only) or both M and F (significant in both sexes where bolded values indicate the sex for which the QTL had the greater effect).

Trait	Ch	Location	Conf. Interval	LOD	*a*	*d*	%	Sex
BMD	1	188.8	185.6–189.3	9.01^†^	**−0.001183**	0.000237	3.73	
	7	124.5	104.1–134.8	4.41^†^	**−0.001229**	−0.000470	3.87	
	8	74.8	67.4–84.5	6.80^†^	**0.001088**	0.000253	2.78	
	9	40.2	34.8–43.4	4.89^†^	**−0.000905**	0.000267	2.26	
	9	82.6	75.9–94.6	6.22^†^	**−0.001032**	−0.000260	2.41	
	10	12.6	9.5–15.8	4.02	**0.000771**	0.000462	1.95	
	12	80.8	78.3–82.9	4.29^†^	**0.000677**	**−0.000620**	1.65	
	13	52.4	46.6–57.8	5.13^†^	**0.000921**	0.000166	2.33	
	15	98.8	96.9–102.0	4.12^†^	**−0.000794**	−0.000220	1.84	M, **F**
	17	31.6	27.7–32.9	8.79^†^	**0.001072**	**−0.000760**	4.23	
	18	41.4	37.6–63.3	3.84	**−0.000616**	**0.000486**	1.53	**M**, F
	X	66.3	58.1–71.5	5.58^†^	**−0.000908**	−0.000038	2.88	
	X	103.5	100.0–124.6	8.50^†^	**−0.001060**	0.000064	3.59	
BMD^a^	1	188.8	185.5–189.3	7.56^†^	**−0.000988**	0.000199	2.60	
	4	140.3	134.2–142.9	4.80^†^	**0.001580**	**0.001921**	10.74	
	8	89.3	72.4–89.3	4.33^†^	**0.000711**	0.000002	1.32	
	9	40.2	34.8–42.1	7.08^†^	**−0.001110**	0.000137	3.03	
	9	59.6	51.4–69.7	4.80^†^	−0.000640	**0.001315**	4.47	
	9	84.0	74.4–93.1	6.29^†^	**−0.000951**	−0.000330	2.00	
	17	31.6	27.3–32.9	6.80^†^	**0.000805**	**−0.000720**	2.69	
	18	24.1	17.0–30.4	3.81	**−0.000689**	0.000405	1.66	
BMC	1	30.8	22.1–35.8	4.59^†^	**0.0118**	0.0025	1.36	
	1	187.0	185.6–189.3	7.71^†^	**−0.0164**	0.0011	2.34	
	2	140.1	132.3–146.2	3.77	**0.0116**	−0.0057	1.77	F
	3	81.6	76.8–90.7	8.33^†^	**0.0131**	**−0.0100**	2.52	
	7	118.6	100.7–134.5	4.21^†^	**−0.0145**	0.0128	2.99	
	8	77.9	70.4–84.5	7.23^†^	**0.0173**	**0.0081**	2.28	
	9	82.6	74.6–96.3	4.56^†^	**−0.0132**	0.0004	1.54	
	10	3.1	3.1–10.8	4.11^†^	**0.0105**	**0.0068**	1.39	
	11	90.3	88.3–92.3	5.17^†^	**0.0124**	**−0.0079**	1.89	M
	12	80.8	78.1–82.9	7.25^†^	**0.0164**	−0.0061	2.65	**M**, F
	12	104.0	91.0–106.6	3.72	**0.0115**	0.0003	1.23	
	13	55.2	54.5–58.1	5.43^†^	**0.0159**	−0.0011	2.01	
	17	26.7	17.9–29.7	5.27^†^	**0.0139**	−0.0066	2.22	
	X	107.7	107.7–118.8	32.16^†^	**−0.0306**	0.0108	9.48	
BMC^a^	1	25.5	21.5–37.7	4.06^†^	**0.0098**	−0.0019	0.91	
	1	187.0	185.5–189.3	6.25^†^	**−0.0122**	−0.0002	1.26	
	3	81.6	76.8–94.3	7.35^†^	**0.0078**	**−0.0105**	1.39	
	4	137.8	126.8–141.6	5.38^†^	**0.0220**	0.0211	6.25	
	9	40.2	34.8–42.1	7.70^†^	**−0.0169**	0.0007	2.27	
	9	81.2	74.6–96.3	4.45^†^	**−0.0114**	−0.0021	1.03	
	X	107.7	107.7–118.8	13.96^†^	**−0.0172**	0.0053	3.02	
BAREA	3	81.6	79.7–82.4	9.89^†^	**0.1946**	−0.0497	3.96	
	12	80.8	75.1–81.9	4.41^†^	**0.1416**	0.0186	1.54	
	X	107.7	107.7–124.7	23.28^†^	**−0.2911**	**0.1118**	6.84	
BAREA^a^	3	81.6	79.7–82.4	7.93^†^	**0.1436**	−0.0666	1.93	
	5	108.8	108.4–113.3	3.93	−0.0558	**0.1681**	1.14	
	X	107.7	107.7–124.7	11.52^†^	**−0.1915**	0.0601	2.98	

### Energy balance traits

Table 2 shows results of the QTL analysis of the eight whole body traits measured in the live F_11_ mice, including those for heat loss (HL) and feed intake (INTAKE), two key energy balance traits. For HL, two QTLs were discovered, one on distal chromosome 1 and another on proximal chromosome 2. Both exhibit additive genetic effects and account for 2.5% and 2%, respectively, of the total variation. A single QTL on chromosome 5 with significant additive effects was found affecting INTAKE.

### Whole body traits

**Figure 1:**
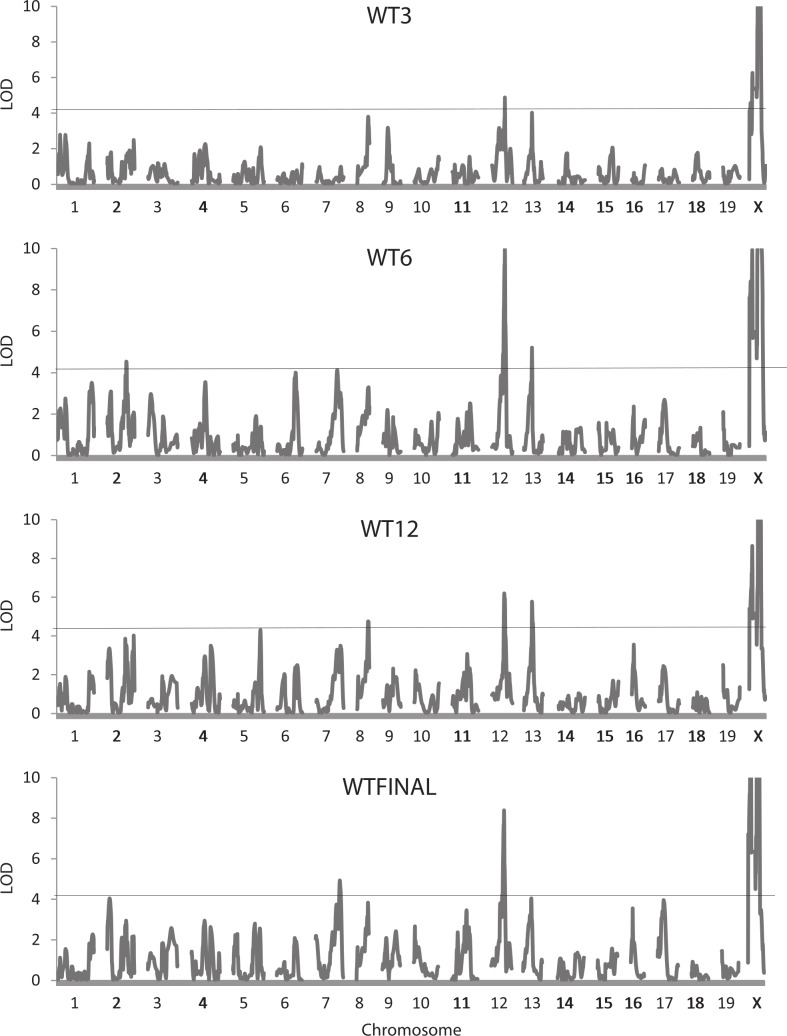
Quantitative trait locus maps of body weight at each of the four ages. Shown are distributions of LOD scores on each of the 20 chromosomes (LOD scores on the X chromosome are truncated to a maximum of 10) for the four weight traits. The horizontal line represents the 95% experimentwise threshold level used in determining statistical significance.

For the whole body traits (all weights, HL, and INTAKE), 37 QTLs were identified, with 27 reaching the 5% experimentwise level of significance (Table 2). LOD scores vary considerably, with the highest values (>20) found for QTLs on the X chromosomes affecting the body weight at 3 (WT3), 6 (WT6), and 12 (WT12) weeks of age and at sacrifice (WTFINAL). Figure 1 illustrates the trends in LOD scores throughout the genome for each of the four body weight traits. The QTLs for the whole body traits are found on 12 of the 20 chromosomes, with chromosome X being most represented (10 occurrences). Confidence intervals for the QTLs range from 3.0 to 24.0 Mb, averaging 12.2 Mb with a standard deviation of 5.97 Mb.

Many of the positions for the QTLs affecting the weight traits are similar. Two QTLs, one on chromosome 12 at 77.0–80.8 Mb, and another on chromosome 13 at 57.4–58.9 Mb, may well represent the same underlying gene with pleiotropic effects on the weight of the mice at each of the four ages. Some QTLs show a more restricted pleiotropy; for example, a QTL on chromosome X at 66.3 Mb affects weight only at the later ages (WT6, WTK12, and WTFINAL), and a potentially common QTL on chromosome 8 (77.9–82.5 Mb) affects WT3, WT12, and WTFINAL (Fig. 1). Additional instances of potential pleiotropy are seen for the four QTLs affecting GAIN3-6, all of which map in similar locations to QTLs for WT6 or WT12. Other QTLs such as the three on chromosome X for WT3 do not exhibit pleiotropy and affect only single traits. This is also the case for the single QTL on chromosome 3 affecting GAIN6-12, the QTL on chromosome 5 affecting INTAKE, and the two QTLs on chromosomes 1 and 2 affecting HL, none of which appear to colocalize with QTLs for any of the other whole body traits.

As evidenced by the significant additive genotypic values for all 37 QTLs affecting the whole body traits (Table 2), they exhibit a predominantly additive mode of action. Significant dominance effects occur for only four QTLs, and the average (absolute) mean of the *d* values (0.21) is well less than that of 0.80 for the *a* values (*d*/*a* ratio = 27%; *P* < 0.01 in a *t*-test for paired data). Dominance is partial or complete for three QTLs although a QTL on chromosome 5 affecting WT12 exhibits overdominance. The percent of the total phenotypic variation in the whole body traits contributed by the QTLs ranges from less than 1% (0.62%) to 5.77%, averaging 1.72%.

**Figure 2:**
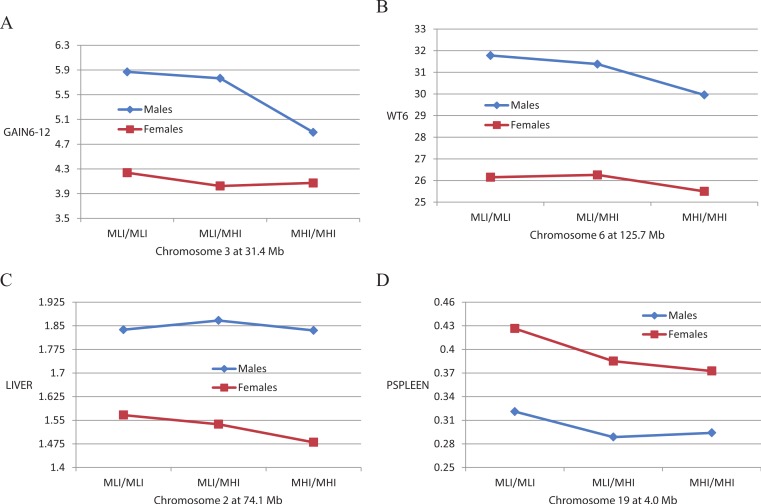
Mean genotypic values of QTLs vary depending on the sex of the mice. Shown are differential effects of the three genotypes (MLI/MLI, MLI/MHI, and MHI/MHI) at QTLs for weight gain from 6 to 12 weeks (GAIN6-12), weight at six weeks (WT6), unadjusted liver weight (LIVER) and spleen weight percentage (PSPLEEN) in male and female mice.

A total of 18 of the 37 QTLs exhibited significant interactions with sex, suggesting that their effects differed in male versus female mice. In 8 of these instances, the QTL effects were significant only in the male mice. An example of this is illustrated in Fig. 2A for a QTL on chromosome 3 affecting GAIN6-12. Note that the means of the three genotypes at this locus in females are quite similar whereas in males, MLI/MLI and MLI/MHI individuals show a greater weight gain than do MHI/MHI individuals. The remaining 10 QTLs show significant effects in both sexes, and in most (8) instances the effect is greater for males. An example of this is illustrated in Fig. 2B for a QTL on chromosome 6 affecting WT6 where trends across the genotypes are similar in both sexes, but are more pronounced in males.

### Body composition traits

**Figure 3:**
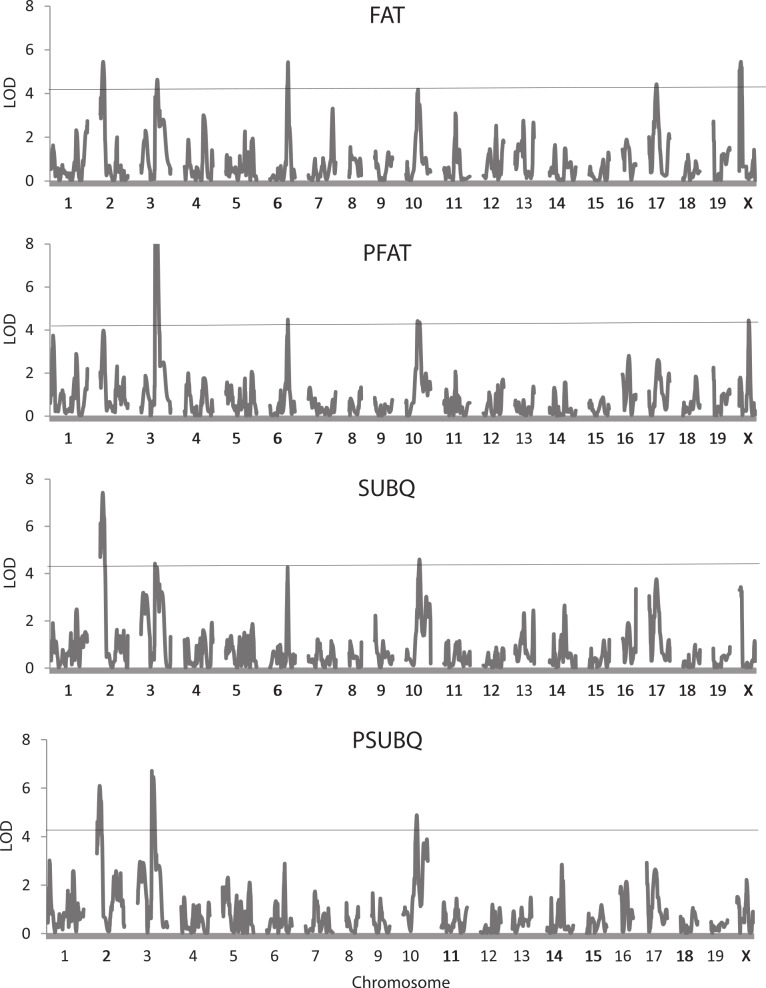
Quantitative trait locus maps of four fat traits. Shown are distributions of LOD scores on each of the 20 chromosomes for FAT, PFAT, SUBQ, and PFAT. The horizontal line represents the 5% experimentwise threshold level used in determining statistical significance.

Table 3 shows results of the QTL analysis of the seven body composition traits adjusted and not adjusted for weight at sacrifice. Figure 3 also illustrates genome trends in LOD scores for two measures of fat, FAT and SUBQ, and their percentages of the final weight (PFAT and PSUBQ). A total of 52 QTLs were found for the body composition traits, 41 of which had LOD scores that exceeded the 5% experimentwise level of significance. These QTLs are found on precisely the same 12 chromosomes as those for the whole body traits already presented in Table 2. Again, chromosome X is the most represented (8 occurrences), although 6 QTLs are found on chromosome 3. Confidence intervals for the QTLs range from 2.5 to 31.6 Mb, averaging 15.3 Mb, somewhat higher than those for the whole body traits. The number of QTLs affecting the body composition traits varies from 0 for PHEART to 7 for PSPLEEN.

In general, there is considerable commonality in the QTLs among the traits and especially between trait pairs. For example, four of the 6 QTLs for FAT and PFAT share similar locations and may well represent the same underlying gene or genes. Similar trends occur for the other trait pairs except for BAT/PBAT, each of which is affected by only one QTL. There is also apparent pleiotropy among the QTLs for different traits such as those on chromosomes 2 (19.4–21.0 Mb) and 3 affecting the FAT/PFAT and SUBQ/PSUBQ traits. Beyond the trait pairs, one or more of QTLs on chromosomes 8, (82.5 Mb), 12 (80.8 Mb), 17 (43.1 Mb) and X (118.9 Mb) affecting WTFINAL (Table 2) also map near those affecting FAT, PFAT, SUBQ, PGON, PBAT, LIVER, PLIV, SPLEEN, and PSPLEEN.

Additive genotypic values are significant for all 52 body composition QTLs, whereas 14 QTLs showed significant dominance genotypic values. Dominance is somewhat more prevalent in these QTLs compared to those for the whole body traits, although the mode of action for the majority of the body composition QTLs is primarily additive. Where significant, dominance is mostly partial or complete, although there are several instances of overdominance (for example, a QTL on chromosome 2 affecting FAT). The percent of the total phenotypic variation in the whole body traits contributed by the QTLs ranges from less than 1% (0.95%) to 7.82%, averaging 2.49%, somewhat higher than the comparable value for the whole body traits.

A total of 11 of the 52 body composition QTLs exhibited significant interactions with sex, a proportion considerably less than that for the QTLs affecting the whole body traits. Two of these QTLs affect females only, an example of which is illustrated in Fig. 2C. This figure shows that a chromosome 2 QTL significantly decreases the mean liver weight of MHI/MHI compared with MLI/MLI female mice, but there is no significant difference in genotype means in male mice. For six other QTLs, five of which affect PSPLEEN, the effect is greater in females than in males. An example of this is illustrated in Fig. 2D where it can be seen that both males and females show the same significant trend in genotypic means for a chromosome 19 QTL affecting PSPLEEN, but this trend is more pronounced in females. Only three QTLs, all affecting GON or PGON, were significant for males only or had greater effects in males, although this trait is different in the two sexes, being a measure of the right epididymal fat pat in males and the perimetrial pad in females.

### Bone traits

**Figure 4:**
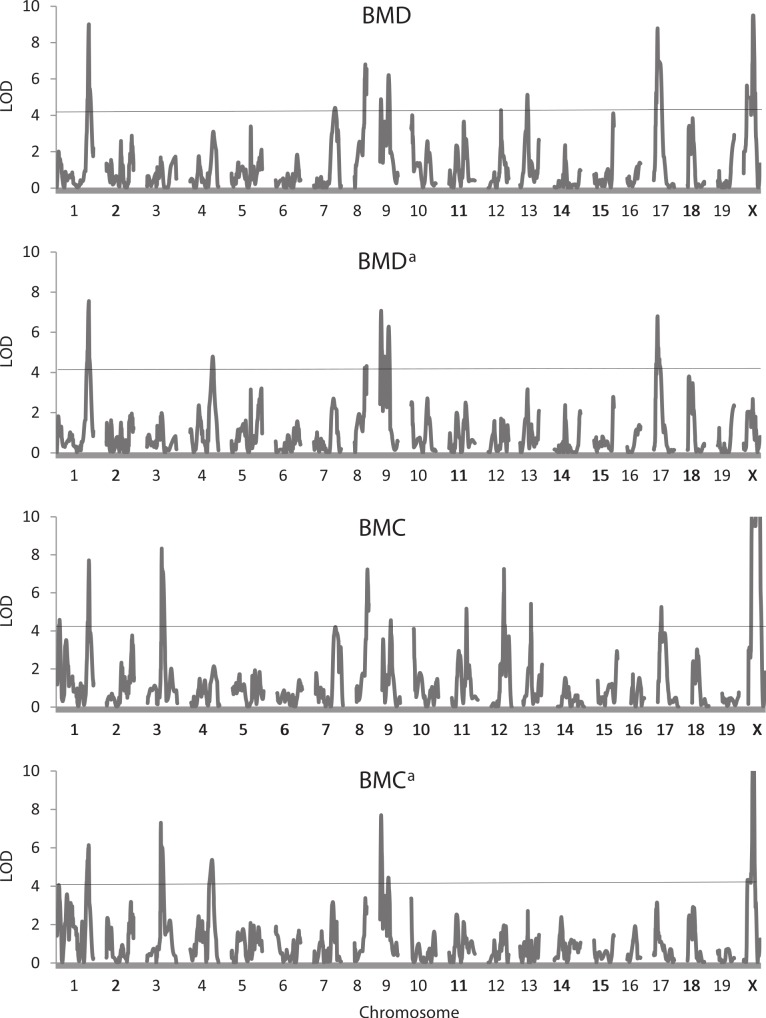
Quantitative trait locus maps of four bone traits. Shown are distributions of LOD scores on each of the 20 chromosomes for BMD, BMD^a^, BMC, and BMC^a^. For BMC and BMC^a^, LOD scores on the X chromosome are truncated to a maximum of 8). The horizontal line represents the 5% experimentwise threshold level used in determining statistical significance.

Table 4 shows results of the QTL analysis of the three bone traits not adjusted and adjusted for weight at sacrifice, and Fig. 4 illustrates trends in LOD scores across the genome for the BMD and BMC trait pairs. A total of 48 QTLs were found for these six traits, 42 exceeding the 5% experimentwise level of significance. The QTLs are found on 16 of the 20 chromosomes, with chromosome 9 (8 occurrences) being most represented. Confidence intervals for the QTLs range from 2.7 to 24.0 Mb, their average of 12.1 being nearly identical to the comparable value for the QTLs affecting the live body traits (Table 2). There is a large number of QTLs affecting the unadjusted BMD (13) and BMC (14) traits whereas QTLs affecting BMD^a^ (8) and BMC^a^ (7) and especially both BAREA and BAREA^a^ (3 each) are fewer in number. A QTL on chromosome 4 has the greatest effect on both BMD^a^ and BMC^a^, although it was not detected for either of the unadjusted bone mineral traits.

Some pleiotropy again is apparent among the QTLs, especially those affecting each pair of traits. For example, QTLs on chromosome 1 (at 199.8 Mb), 9 (at 40.2 and 82.6–84 Mb) and 17 (at 31.6 Mb) affect both BMD and BMD^a^. Some QTLs also appear to be common across traits, an example being one on chromosome 1 at 187–188.8 Mb affecting both the adjusted and unadjusted BMD and BMC traits. Again, at least four of the QTLs affecting WTFINAL (those on chromosomes 8, 11, 12, 13) map to similar or identical positions as those affecting one or more of the bone traits.

All except 2 of the 48 QTLs affecting the bone traits show significant additive genotypic effects, with the mean of their absolute values = 0.028. Significant dominance effects occur for 13 QTLs, with the mean of the absolute *d* values = 0.013 (mean *d*/*a* ratio = 0.49). Most of the dominance tends to be partial or complete, with only two clear instances of overdominance (one QTL on chromosome 9 at 59.6 Mb affecting BMD^a^, and another QTL on chromosome 5 at 108.8 Mb affecting BAREA^a^). The percentage of the total phenotypic variation in the bone traits contributed by the QTLs ranges from 0.91% to 10.7%, and averages 2.78%.

Only five of the QTLs for the bone traits exhibited sex interactions, so the effects of the majority of these QTLs were consistent in both sexes. Further, all five QTLs showing interactions occurred for BMD or BMC, not the adjusted values for these traits (BMD^a^ and BMC^a^) or for either the raw or adjusted BAREA traits.

**Figure 5:**
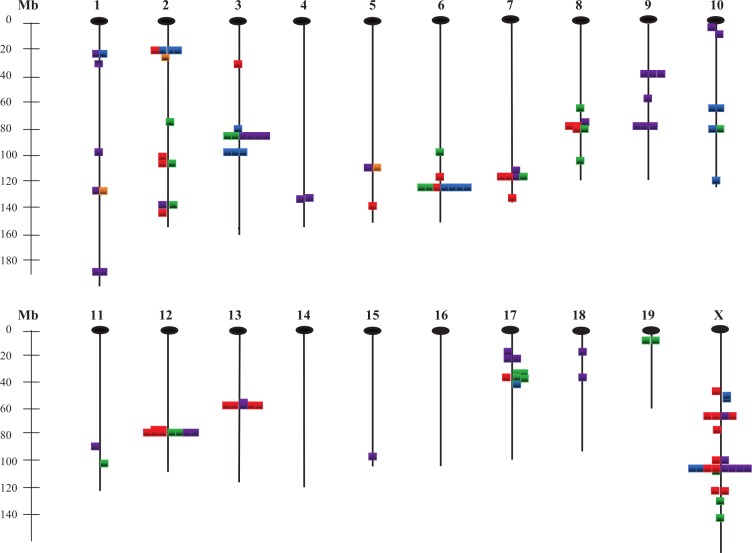
Chromosomal locations of QTLs affecting the 28 measured traits. Gold, energy intake/expenditure traits; red, body weight traits; blue, adiposity traits; green, organ weight traits; purple, bone traits; Mb, mega base pairs.

Figure 5 illustrates the locations of all QTLs found for the 28 traits, with different colors (see legend) representing the various trait groups. Although there are some QTLs in unique positions that affect only one trait, most are located in clusters where several traits are affected. This apparent pleiotropy is particularly noticeable for QTLs affecting traits within trait groups, but in some instances occurs across groups as well.

## Discussion

We undertook this study to search for QTLs affecting energy balance traits in a unique F_11_ mouse population derived from an intercross of lines that had undergone long-term divergent selection for heat loss measured by indirect calorimetry. An important goal was to uncover QTLs for heat loss itself, and to discover whether they might be commonly affecting other traits, especially measures of fat. We did find two QTLs for HL, although expected more given the history of the F_11_ population.

**Table 5:** Adiposity QTLs and their potential candidate genes. Shown are the chromosome (Chrom) and location of the QTLs affecting the various adiposity traits, as well as the number of protein-coding genes located within their confidence intervals, and potential candidate genes for the QTLs.

Chrom	Location (Mb)	Adiposity traits	No. of genes	Candidate genes
1	21.4–35.8	PFAT	47	*Rims1*, *Arhgef4*
2	11.5–29.4	FAT, PFAT, SUBQ, PSUBQ	339	*Cacna1b*, *Ehnt1, Cel*
3	79.5–114.7	FAT, PFAT, SUBQ, PSUBQ, PGON	511	*Prkab2*, *Nhlh2*, *Kcna3*
6	117.4–126.6	FAT, PFAT, SUBQ, GON, PGON, BAT	134	*Ankrd26*, *Adipor2*, *Gdf3*,
10	65.0–100.0	FAT, PFAT, SUBQ, PSUBQ	395	*Arid5b, Igf1*
10	114.0–130.3	PSUBQ	225	*Hmga2, Lrp1, Mmp19*
17	34.9–62.7	FAT, SUBQ	397	*Ehmt2, Lta, Tnf, Rcan2*
X	48.3–66.5	FAT, GON	86	*Gpc3, Hprt, Brs3*
X	101.5–130.5	PFAT, PGON, PBAT	113	*Cited1*

Among the 28 traits, however, we were successful in uncovering a total of 137 QTLs that were located at various sites on all chromosomes except 14 and 16. QTLs were found for all traits except PHEART, and the number affecting these traits varied from only 1 (GAIN6-12, INTAKE, and BAT) to as many as 14 (BMC). A major finding was that the X chromosome harbored the greatest number of these QTLs as well as the QTLs with the greatest effects on many phenotypes.

Leamy et al. (2005) previously showed that there were a number of significant genetic correlations among the traits in this F_11_ population, so it was not surprising that many of the QTLs we found exhibited apparent pleiotropy. As a consequence, a number of the 137 QTLs presumably represent common underlying genetic variation. In fact a tally of all non-overlapping confidence intervals for these QTLs suggests that they may reside in as few as 41 unique genomic locations. This number is conservative since more sites presumably would emerge with an increase in mapping precision, but in general the precision of the QTLs as assessed by the mean of their confidence intervals, 13.3 Mb, was quite good. This value is comparable to that of 12.5 Mb estimated for QTL confidence intervals affecting similar traits in an F_10_ advanced intercross mouse population analyzed by Leamy et al. (2012) and Leamy et al. (2013), and well below that of 23 Mb calculated by Kelly et al. (2011) for comparable traits in an F_4_ advanced intercross mouse population.

Below we present a detailed discussion of the results of the QTL analyses for each of the 28 traits, first considering those QTLs affecting the two key energy consumption and expenditure traits, HL and INTAKE. We then follow with a discussion of QTLs discovered for the body weight, adiposity, organ weight, and bone traits. Traits within each of these four groups are generally moderately to highly correlated, and as was shown in the results above, often tended to be affected by common QTLs (pleiotropy).

### Energy consumption and expenditure QTLs

The two QTLs we discovered for HL were far fewer than the nine QTLs found for this same trait by Moody et al. (1999) in their HB (high heat loss selection line crossed with C57BL/6J) mouse population, and also mapped to different positions. Moody et al. (1999) tested whether the three QTLs (one on chromosome 1 and two on chromosome 3) exhibiting the greatest effect on heat loss in the HB population would replicate in an F_2_ population (LH) created from crossing mice from the outbred lines they had selected for increased (MH) and decreased heat loss (ML). The single QTL on chromosome 1 was confirmed, although with a considerably reduced effect, and neither QTL on chromosome 3 replicated (Moody et al., 1999). The HB and LH populations both share an LH progenitor, so this disparity in results presumably reflected differences in the alleles segregating in the low heat loss lines (BL and ML) and/or in the interactions of alleles on the two separate genetic backgrounds (Moody et al., 1999). These differences also help to explain why the F_11_ advanced intercross population produced from inbreeding and crossing of mice in the selection lines has yielded QTLs for HL that differ in number and location from those originally found in the HB population. It is also possible the alleles at heat loss QTL that were still segregating in the selection lines were lost during the inbreeding process, or that they were not well represented in the specific mating pairs leading to these inbred lines.

It is interesting that the HL QTL we discovered on chromosome 1 (128.7 Mb) maps near a QTL on this same chromosome (at 127.2) that affects spleen weight (both SPLEEN and PSPLEEN). Although we cannot know whether there is a single gene underlying these QTLs that in fact is pleiotropically affecting both HL and SPLEEN, it is certainly possible given that the genetic correlation of these two traits estimated by Leamy et al. (2005) is a moderately high +0.48. This also seems reasonable because mice in the MH selection group tended to have larger spleens than those in the ML line, this presumably being a reflection of their greater energy consumption and expenditure (Moody, Pomp & Nielsen, 1997). In addition, the spleen is a high metabolic rate organ that has been shown to make important contributions to resting energy expenditure in humans (Javed et al., 2010). The greatest number of QTLs among the body composition traits were found for the spleen traits (SPLEEN and PSPLEEN), and perhaps some of these other QTLs may be involved with energy balance as well. If so, selection for spleen weight may be an efficient alternative to produce lines divergent for energy balance.

The other heat loss QTL that we discovered on chromosome 2 (at 25.6 Mb) maps within the confidence intervals of QTLs for two adiposity traits: FAT and SUBQ (also PFAT and PSUBQ). Moody et al. (1999) found a similar result for 4 of 9 HL QTLs in their HB mouse population. Thus it seems possible that our chromosome 2 HL QTL may have pleiotropic effects on both heat loss and adiposity. Genetic correlations of HL with the four adjusted and unadjusted adiposity traits in the F_11_ mouse population all are less than |0.2| and are non-significant (Leamy et al., 2005), so it is not surprising that we did not find more evidence for this sort of pleiotropy. While the nature of this chromosome 2 QTL is unknown, *Pax8*, *paired box gene 8* at 24.4 Mb (Planchov et al., 1990) is an interesting possibility for a candidate gene that could affect both heat loss and adiposity. Mutations in *Pax8* cause hypothyroidism with its consequent effects on metabolism and growth that have been documented in mice (Planchov et al., 1990) and in humans (Trueba et al., 2005).

We found just one QTL on chromosome 5 affecting feed intake (INTAKE), the primary measure of energy consumption. This seems surprising given that by generation 15, Nielsen et al. (1997b) achieved a nearly 21% divergence in feed intake between the high and low selection lines from which the F_11_ population was generated. And Leamy et al. (2005) found a fairly low, but significant heritability of 0.27 for this trait in the F_11_ population. On the other hand, Moody et al. (1999) found no QTLs for feed intake in the HB F_2_ mouse population. So perhaps it is understandable that we found little detectable genetic (QTL) variation for this trait in our F_11_ mouse population. However, studies in other mouse populations have yielded QTLs for feed intake on several different autosomes (Allan, Eisen & Pomp, 2005; Leamy et al., 2012) and on the X chromosome (Liu et al., 2001).

### Body weight QTLs

The pleiotropic patterns exhibited by the QTLs affecting body weight at each of the four ages were consistent with those reported in previous mouse QTL studies (Cheverud et al., 1996; Vaughn et al., 1999; Rocha et al., 2004; Gordon et al., 2008). For example, Cheverud et al. (1996) found QTLs affecting early growth in mice (body weight from weeks 1 to 3) that were distinct from those affecting later growth (body weight from weeks 6 to 10), but some QTLs that affected both early and late growth. We also found QTLs (on chromosomes 8, 12, and 13) affecting both early (WT3) and late growth (WT6, WT12, WTFINAL), as well as other QTLs affecting early growth only, late growth only, or body weight at a single age. In all cases the additive genetic effects of those QTLs exhibiting pleiotropy were consistent in sign but tended to increase in magnitude from early to late growth, as also is typical (Cheverud et al., 1996; Vaughn et al., 1999).

It was somewhat surprising to find so many X-linked QTLs that tended to exhibit the highest LOD scores and contributions to the total variation of the body weight traits. This sort of result has not usually been found (for example Leamy, Pomp & Lightfoot, 2009b; Leamy et al., 2012), but mapping information for the X chromosome is somewhat more limited because many previous mouse studies have analyzed only the 19 autosomes (Cheverud et al., 1996; Cheverud et al., 2011; Vaughn et al., 1999; Rocha et al., 2004; Gordon et al., 2008; Kelly et al., 2011; Leamy et al., 2012). Some body weight and adiposity QTLs have been found on this chromosome (Dragani et al., 1995; Rance, Hill & Keightley, 1997), many of which are listed in the Mouse Genome Database (2013). In addition, some of the previously mapped X-linked QTLs have very strong effects. However, none of these appear to map in similar positions to those we have found, and this may be a consequence of the imprecision of mapping or instead suggest that the X-linked body weight QTLs we have uncovered may be novel. Since no QTLs for heat loss were mapped to the X chromosome, it seems unlikely that these QTLs played a role in the selection response observed in the MH and ML lines, but rather that they represent variability segregating in the base population from which selection originated.

Nearly one-half (14) of the 29 body weight QTLs showed significant interactions with sex, with all except two affecting males only or showing greater effects in males. Although a few previous studies in mice have failed to detect QTL by sex interactions for body weight (Rocha et al., 2004; Leamy et al., 2012), these kinds of interactions are quite common in other studies (Vaughn et al., 1999; Cheverud et al., 2001; Cheverud et al., 2011; Gordon et al., 2008). Some studies also have shown that a preponderance of body weight QTLs affect male rather than female mice (Dragani et al., 1995; Vaughn et al., 1999). Significant interactions of sex with epistatic (two-locus) QTL effects also have been found (Leamy, Gordon & Pomp, 2011), and it is possible that these may have modified or even masked the effect of some of the body weight QTLs in the female F_11_ mice. Whatever the physiological mechanism involved in these differential QTL effects, they are an important component of the genetic architecture of body weight.

We can only speculate about the identity of the body weight QTLs because their confidence intervals usually include a number of potential candidate genes, even with the finer resolution afforded by an AIL. For example, the Mouse Genome Database (2013) lists 38 protein coding genes even in the smallest (3 Mb) confidence interval found for any body weight QTL (one on the X chromosome at 101.4 Mb affecting WT3). This kind of result is typical in QTL mapping experiments and has made the transition from QTL to gene difficult, although some progress is being made. For example, Oliver et al. (2005) fine-mapped an X-linked growth QTL to a small region containing *Gpc3* (*glypican 3*), and provided strong evidence from expression data for this being the gene underlying the QTL. *Gpc3* actually is within the confidence interval of a QTL we discovered on chromosome X at 50.4 Mb affecting WT3, and thus represents a potential candidate gene for this QTL as well. If we have mapped this same gene, however, its effect on body weight in the F_11_ mice is much smaller than was previously found in other mouse populations (Liu, Bunger & Keightley, 2001).

### Adiposity QTLs

We found a total of 28 QTLs affecting the adiposity traits that are located in nine non-overlapping regions on seven different chromosomes, including one QTL on each of chromosomes 1, 2, 3, 6, and 17, and two on each on chromosomes 10 and X (see Table 5). Only the QTL on chromosome 17 matches any of the QTLs found by Moody et al. (1999) for these same adiposity traits in their MB mouse population. Other QTLs for various measures of adiposity previously have been mapped near those we have discovered on chromosomes 2 and 10 (Mouse Genome Database, 2013) as well, but not those on chromosomes 1, 3, 6, and X. These 5 obesity QTL sites therefore may be unique, and add to the current total of 170 obesity QTL locations listed in the Mouse Genome Database (2013).

Among the nine adiposity QTLs we discovered, seven affected two or more adiposity traits whereas only two were trait-specific. We expected this high level of pleiotropy because genetic correlations previously calculated among the four traits all were positive and quite high, varying from +0.71 to +0.95 (Leamy et al., 2005). We also found considerable commonality among the QTLs affecting the adjusted/unadjusted trait pairs. Of the six QTLs affecting FAT for example, four replicated with PFAT and two (on chromosomes 2 and X) did not. Further, both non-replicating QTLs affected at least one other adiposity trait, so they were not unique to FAT. Only two QTLs on chromosomes 1 and 10 affected one trait, and it is noteworthy that both had LOD scores reaching the suggestive, but not significant, experimentwise threshold. Nonetheless, the differences among the QTLs affecting the trait pairs are sufficient to suggest caution in comparing QTL results for unadjusted versus adjusted trait values.

Four adiposity QTLs (on each on chromosomes 6 and 17, and two on chromosome X) also mapped in the same general locations as QTLs affecting one or more of the body weight traits. This apparent pleiotropy for QTLs affecting both body weight and adiposity traits is not uncommon, even when adiposity measures have been adjusted for overall body size (Cheverud et al., 2001; Kelly et al., 2011; Leamy et al., 2012). The adiposity QTL on distal chromosome X mapped to a similar location for QTLs affecting GAIN3-6, WT6, WT12 and WTFINAL. If common, this gene appears to influence body weight in mice from six weeks of age until the time of sacrifice, as well as adiposity measured at that time. The additive genotypic values of this potentially common QTL, however, is consistently positive for the adiposity traits but negative for the body weight traits, suggesting that it is exhibiting antagonistic pleiotropy.

Although many protein coding genes fall within the confidence intervals of the obesity QTLs we discovered, we used the Mouse Genome Database (2013) and found some potential candidates for each of the QTLs (Table 5). For example, all three genes listed as candidates for the obesity QTL on chromosome 6 have well documented effects on adiposity, metabolism, and homeostasis in mice (Bera et al., 2008; Bjursell et al., 2007; Shen et al., 2009). Similarly, *Brs3, bombesin-like receptor 3*, at 57 Mb on chromosome X, is a possible candidate for our proximal X obesity QTL since mice with mutations at this locus exhibit obesity, an impaired glucose metabolism, and a reduced metabolic rate (Ladenheim et al., 2008). Interestingly, Xu et al. (2012) have shown that *Brs3* shows a sexually dimorphic expression in the hypothalamus that apparently is a reflection of its role in the regulation of sex typical behaviors in mice. For the other (more distal) X-linked obesity QTL, we found only one potential candidate: *Cited1* (at 102.2 Mb). Alterations in this gene are associated with an increased incidence of diabetes and obesity in mice (Novitskaya, Baserga & de Caestecker, 2011).

### Organ weight QTLs

We found several QTLs affecting liver, heart, and especially spleen weights in the F_11_ mice. This was as expected since liver and spleen weights significantly differed between the heat loss selection lines (Moody, Pomp & Nielsen, 1997) from which the F_11_ population was derived. Moody et al. (1999) uncovered 5 QTLs for the percentage of liver weight in their MB mouse population, but only one of these on chromosome 7 maps within the confidence interval of a QTL we discovered for LIVER. They also found two QTLs on chromosomes 1 and 7 affecting the heart weight percentage but we found none for PHEART.

The QTLs for the organ weights mostly were independent from those we found for the adiposity QTLs. The only exceptions were QTLs on chromosome 17 affecting LIVER, SPLEEN, and PSPLEEN and an X-linked QTL affecting PLIV and PSPLEEN, both of which mapped within the confidence intervals of adiposity QTLs. It is interesting that the QTL on chromosome 17 affected LIVER but not PLIVER, suggesting it may have pleiotropic effects on overall body size, and in fact it also maps in a similar location to a QTL for WTFINAL. Of the candidate genes listed for this chromosome 17 adiposity QTL (Table 5), *Rcan2* is attractive because alterations in this gene reduce diet-induced obesity and liver weight (Sun et al., 2011). The QTL on chromosome X affects PLIVER and not LIVER, and may be the same as the QTL previously described possibly exhibiting antagonistic pleiotropic effects on the body weights versus the adiposity traits.

We found four QTLs on chromosomes 1, 8, 12, and 17 for SPLEEN, all of which were replicated for PSPLEEN. Of these QTLs, only that on chromosome 17 mapped close to one for LIVER, and may well represent the same QTL described above. A QTL on chromosome 12 (at 81.9 Mb) had the greatest effect on spleen weight. *Psen1*, *presenilin 1* (at 83.6 Mb), is a potential candidate gene for this QTL since when altered it can cause many effects, including enlargement of the spleen (De Strooper et al., 1998). Another possibility is *Ucp1*, *uncoupling protein 1* (at 83.3 Mb), that affects thermoregulation and brown fat development (Jacobsson et al., 1995) , but when knocked out also causes a reduction in spleen cell numbers (Adams, Kelly & Porter, 2010).

It was interesting that five of the seven PSPLEEN QTLs, but no SPLEEN QTLs, exhibited significant interactions with sex. This disparity may be a simple consequence of scaling a small organ weight by overall body weight, however, and in fact preliminary analyses of variance showed a much greater sexual dimorphism for PSPLEEN than for SPLEEN. Further, the differential effects of the QTLs in males versus females, one example of which was previously illustrated in Fig. 1, were rather subtle. Nonetheless, this interaction occurred even for two X-linked QTLs, including one at 136.6 Mb that appears to be unique. A possible candidate for this QTL is *Sty14* (at 133.9), a gene that exhibits sexually dimorphic expression in the brain (Xu et al., 2012). Inactivation of this gene causes an increase in adrenocorticotropin secretion that in turn is known to alter spleen weights (Veenema et al., 2003).

### Bone QTLs

Heritability estimates among the traits analyzed by Leamy et al. (2005) in the F_11_ mouse population were highest for the unadjusted BMD (0.65) and especially BMC traits (0.85), and thus it was not surprising that we also found the greatest number of QTLs for these two traits (13 for BMD, 14 for BMC) as well. BAREA had a much lower heritability (0.26), and we found only 3 QTLs affecting this trait. In fact across all traits, there is a significant positive association (Spearman correlation = + 0.74, *P* < 0.01) between the number of QTLs affecting the traits and their heritabilities. Adjusting BMD and BMC traits for WTFINAL reduced the number of QTLs by about one-half, however, suggesting that at least some of these QTLs were influencing overall growth. And in fact 7 of the QTLs for both BMD and BMC mapped close to those affecting the body weights, especially WTFINAL. Two QTLs (both X-linked) for BMD and three QTLs for BMC also mapped near QTLs for one or more adiposity traits.

QTLs on chromosome 9 were important contributors to the adjusted bone mineral traits, three affecting BMD^a^ and two affecting BMC^a^. A possible candidate for the most proximal QTL at 40.2 Mb on this chromosome affecting both of these traits is *Zfp202*, *zinc finger protein*, *202*. This gene also is located at 40.2 Mb, and when altered causes abnormal bone mineralization (Mouse Genome Database, 2013). The most distal chromosome 9 QTL (84.0 and 81.2 Mb) affecting both BMD^a^ and BMC^a^ may be *Col12a1*, *collagen, type XII*, *alpha 1* at 79.6 Mb. Mutations in this gene produce lower mineral apposition rates and a reduced mineralized surface/total bone surface (Izu et al., 2011). Leamy et al. (2013) also found two QTLs at similar locations on chromosome 9 (35.0 and 82.9 Mb) affecting total bone mineral density in their F_10_ advanced intercross population. Another QTL with an intermediate location (59.6 Mb) on chromosome 9 affected BMD^a^ in the F_11_ mice, and a possible candidate is *Glce*, *glucuronyl C5-epimerase* (at 62.1 Mb), mutations in which lead to excessive bone mineralization (Li et al., 2003).

Except for an additional QTL on chromosome 9 affecting BMD^a^, only one QTL was found for the adjusted bone mineral traits that was not detected for the unadjusted bone mineral traits. This QTL on chromosome 4 (at 140.3 and 137.8) also had the greatest effect on these traits, accounting for nearly 11% of the total variance for BMD^a^. This value may be inflated, however, because this QTL occurs in a region where markers are very sparse. Others also have mapped QTLs for bone mineral density in this region (Klein et al., 2001; Masinde et al., 2002; Koller et al., 2003).

## Summary

In summary, we conducted an extensive genome-wide scan for a wide variety of metabolism traits within an advanced intercross line derived from lines divergently selected for heat loss. While only two QTLs for heat loss were detected, we uncovered a total of 137 QTLs at 41 unique sites on 18 of the 20 chromosomes in the mouse genome, with X-linked QTLs being most prevalent and having the strongest effects. The number of QTLs affecting the various traits generally was consistent with previous estimates of heritabilities in the same population, with the most found for two bone mineral traits and the least for feed intake and several body composition traits. QTLs were generally additive in their effects, and some, especially those affecting the body weight traits, were sex-specific. Pleiotropy was extensive within trait groups (body weights, adiposity and organ weight traits, bone traits) and especially between body composition traits adjusted and not adjusted for body weight at sacrifice. Not all QTLs affecting each of the trait pairs adjusted/not adjusted for body weight (FAT, PFAT, etc.) were identical, however, and we were able to identify a number of unique QTLs affecting the (adjusted) traits independently of overall body size. This study, combining QTL mapping with genetic parameter analysis in a large segregating population, advances our understanding of the genetic architecture of complex traits related to obesity.

## Supplemental Information

10.7717/peerj.392/supp-1 Appendix S1 SNP markers used with their chromosome and coordinates

10.7717/peerj.392/supp-2 Appendix S2 MLI/MLI, MHI/MHI, and MLI/MHI genotype frequencies at each marker on all chromosomes Mb = megabases.

10.7717/peerj.392/supp-3 Supplemental Information 3 Example of QTLRel code
