# Supplementary material for: Quantitative trait loci for energy balance traits in an advanced intercross line derived from mice divergently selected for heat loss
Source: PeerJ. 2014 May 27;2:e392. doi: 10.7717/peerj.392 (PMC4045330; doi:10.7717/peerj.392)
Supplement: Appendix S1 [file peerj-02-392-s001.pdf]

Marker	Chromosome	Coordinate	Marker	Chromosome	Coordinate
rs3677817	1	5.197303	rs13476089	1	122.457718
rs13475712	1	7.41187	rs3697826	1	124.896697
rs3726952	1	12.906283	rs13476098	1	126.400694
rs13475735	1	15.147784	rs6355835	1	130.487043
rs3658044	1	19.498836	rs3713473	1	131.948608
rs3711079	1	22.156681	rs3700475	1	138.09948
rs4222215	1	24.234159	CEL-1_140588762	1	140.588762
rs13475771	1	25.676777	rs13476147	1	142.551146
rs3677683	1	27.510356	rs6186115	1	144.615698
rs6237824	1	30.982111	rs6364156	1	146.112228
UT_1_35.224766	1	34.822105	gnf01.149.342	1	149.831572
rs13475816	1	37.880203	rs13476187	1	154.18791
rs13475821	1	39.844439	rs8242852	1	171.128307
mCV23591750	1	51.617463	rs3143355	1	175.96311
rs8254826	1	61.776437	rs13476259	1	177.399967
rs3716105	1	61.943426	rs6301437	1	179.84146
rs6356603	1	75.36232	CEL-1_181947877	1	181.947877
rs6321468	1	78.484909	rs3693165	1	183.213131
rs3667200	1	78.622208	rs6154379	1	184.815448
rs13475972	1	89.01772	rs13476290	1	186.816908
rs13475960	1	89.120328	gnf01.195.387	1	191.348177
rs6250696	1	89.196129	rs4222922	1	193.166401
rs13475973	1	89.403365	rs6246360	1	194.9579
gnf01.089.691	1	89.659368	rs13476318	2	3.076675
rs3022827	1	90.567434	rs13476330	2	5.79421
rs13475982	1	92.258955	rs6240512	2	10.929543
rs13475988	1	93.553382	rs13476352	2	13.387323
rs13475989	1	93.819709	rs8250941	2	25.427137
rs13475991	1	94.403364	rs3718405	2	27.249691
rs3675505	1	95.019059	rs6181760	2	27.847598
rs6342650	1	96.400818	rs13476429	2	35.357114
rs6358447	1	97.506398	rs13476553	2	66.906537
rs13476003	1	97.677389	rs13476556	2	67.617045
CEL-1_98681809	1	98.681809	rs6371268	2	68.927682
CEL-1_98799654	1	98.799654	rs13476560	2	69.403391
gnf01.099.019	1	99.03077	rs13476563	2	70.070631
rs3695980	1	99.464143	rs3682843	2	71.050924
rs13476012	1	99.797518	rs3670752	2	71.26882
rs13476014	1	100.146917	rs3683059	2	75.399681
rs3685663	1	100.268247	rs6248415	2	76.480252
rs3717264	1	103.113538	mCV25095764	2	76.990271
rs3664662	1	104.472398	rs3711780	2	77.245946
rs3664301	1	107.059245	CEL-2_79237503	2	79.237503
rs3685919	1	109.634975	rs13476594	2	79.662978
rs3725409	1	116.434018	rs3722345	2	80.509747
rs3695581	1	118.500635	rs13476639	2	92.720804
rs3667720	1	120.697988	rs4223268	2	93.313855

rs13476663	2	99.952945	rs6224355	3	66.3279
CEL-2_100344390	2	100.34439	rs13477165	3	66.436519
rs6249987	2	100.974556	rs6226544	3	67.856811
rs13476667	2	101.477932	CEL-3_68001820	3	68.00182
rs13476669	2	101.877243	rs13477178	3	70.089276
rs6155648	2	102.059025	rs6198234	3	70.36143
rs3700286	2	102.525512	CEL-3_70552044	3	70.552044
rs3143810	2	103.177373	CEL-3_70697605	3	70.697605
rs3674721	2	104.594142	rs3698109	3	71.209119
mCV25337624	2	105.399199	rs6264454	3	71.858927
rs13476684	2	105.859705	rs3715136	3	72.730362
rs13476689	2	107.356662	rs13477190	3	73.392003
rs13476693	2	109.285116	rs13477210	3	77.646357
rs3022892	2	109.891529	rs3715352	3	78.618892
rs13476697	2	110.552699	rs13477215	3	78.622271
rs13476700	2	111.672465	rs3659866	3	81.285486
rs3693678	2	112.367495	rs6305129	3	81.508202
rs3701250	2	114.662573	gnf03.079.138	3	82.509078
rs6276129	2	116.113235	rs13477233	3	83.880528
rs3677413	2	116.603199	rs3708227	3	84.422484
rs3723406	2	117.650393	rs6376008	3	87.149248
rs13476728	2	118.595838	rs13477244	3	87.394444
rs6340352	2	121.051368	rs3722681	3	110.165817
rs13476746	2	122.660089	rs6242665	3	111.412955
rs13476755	2	124.470148	rs6214597	3	117.350554
rs3697020	2	125.287975	rs13477498	3	153.484447
rs6411422	2	128.122514	rs6331755	3	156.678486
rs6161193	2	129.466095	rs3667025	3	158.614152
rs3699051	2	132.104826	rs13477528	3	160.447976
rs13476788	2	135.061275	rs13477534	4	4.046388
rs13476794	2	136.490987	rs13477546	4	7.682681
rs3710324	2	136.973991	rs13477592	4	19.653411
rs13476805	2	139.473083	rs13477599	4	21.606001
rs6303304	2	141.192338	rs13477617	4	27.105003
rs6360457	2	141.929558	rs13477637	4	33.888067
gnf02.141.261	2	142.837835	rs13477662	4	39.882315
rs6195594	2	143.346715	CZECH-4_46713961	4	46.713961
rs3696870	2	147.404525	rs3676423	4	51.03541
rs13476827	2	148.284246	UT_4_57.645957	4	57.161636
rs3676033	2	149.197838	rs13477735	4	60.185456
rs3719352	3	10.752146	rs13477741	4	62.556359
rs3694133	3	12.562659	CEL-4_74066970	4	74.06697
rs6398851	3	14.447458	rs6258088	4	80.891223
rs13476992	3	16.59583	rs13477813	4	82.905304
rs13477043	3	31.257023	rs3726736	4	102.512069
rs13477046	3	32.50179	rs13477895	4	104.999653
rs6363066	3	57.943185	rs3695162	4	107.621073
rs6239288	3	60.673679	rs3670382	4	109.18677
rs3696955	3	63.295533			

rs3694396	4	112.812399	rs13478521	5	125.897723
rs3696331	4	115.756451	rs3661159	5	128.187011
rs3726907	4	118.140601	rs13478546	5	133.978719
gnf04.117.960	4	120.989378	rs6334078	5	136.117818
rs3671259	4	123.272188	rs6319445	5	139.022464
rs13477972	4	126.623471	rs3023061	5	141.390786
rs6268364	4	149.902058	rs6191249	5	143.668186
rs3693138	4	152.591465	rs3668534	5	145.8327
rs3693087	4	154.007691	rs3692702	5	147.670924
rs13478092	5	3.601413	rs3655269	6	17.7151
rs13478104	5	7.984264	rs13478656	6	21.755667
rs3714258	5	11.417181	rs3684860	6	48.907491
CEL-5_14611794	5	14.611794	rs3023069	6	52.190471
gnf05.014.723	5	19.14048	rs13478761	6	53.702681
rs13478133	5	19.993162	rs6215332	6	60.960747
UT_5_19.849706	5	20.127688	mhcCD8b4	6	71.532444
rs13478136	5	20.633475	rs3672029	6	75.631345
rs13478138	5	21.021884	rs13478841	6	78.477103
rs13478145	5	23.049118	rs3698364	6	81.140583
rs6349956	5	23.273638	rs6268125	6	87.214811
rs3706626	5	23.524916	rs13478891	6	92.361613
CEL-5_24211033	5	24.211033	rs6292642	6	104.934072
rs13478151	5	24.590157	rs3655148	6	108.183645
rs3705209	5	24.811094	rs6204829	6	116.303467
rs3699500	5	25.485429	rs13478997	6	118.461935
rs3668113	5	26.583853	rs3695724	6	120.403722
rs13478157	5	26.724567	CEL-6_122563022	6	122.563022
rs3680434	5	27.162034	gnf06.122.747	6	124.630688
rs3682333	5	30.357756	rs3670851	6	129.225234
UT_5_30.642219	5	30.705556	rs6339546	6	134.019679
rs13459083	5	30.900782	rs8268650	6	141.660661
rs3700706	5	31.071103	rs6283083	6	144.006253
rs6408534	5	33.877338	rs6387265	6	145.879903
CEL-5_34263494	5	34.263494	rs13479092	6	147.7388
rs3716195	5	40.684934	CEL-7_6502564	7	6.502564
rs6276465	5	40.835483	rs13479140	7	8.646169
rs13478210	5	41.410481	rs13479163	7	16.00115
rs13478212	5	41.787529	mCV25220583	7	17.994561
rs13478215	5	42.528407	rs4226520	7	18.75874
mCV27558149	5	64.412079	gnf07.032.360	7	27.852716
gnf05.061.650	5	65.952319	gnf07.032.889	7	28.379711
rs3722245	5	74.543251	CEL-7_29429804	7	29.429804
rs13478428	5	99.993018	rs6313526	7	32.302462
rs4225398	5	105.084101	rs3703247	7	33.404727
rs6224339	5	110.516973	rs6295036	7	33.671286
rs3663141	5	114.196024	rs13479234	7	35.506778
mCV25130934	5	117.090128	CEL-7_36545579	7	36.545579
CEL-5_120064766	5	120.064766	rs13479238	7	36.904642
rs13478508	5	122.876611	rs13479258	7	41.532185

gnf07.050.858	7	44.11275	rs13480285	9	76.908789
rs6388842	7	44.912991	rs13480308	9	83.133092
mCV23672419	7	45.173762	rs13480317	9	86.214826
rs3693038	7	45.306964	rs6309331	9	92.107776
rs13479274	7	45.712494	rs3725272	9	95.000478
rs13479276	7	46.065034	rs13480364	9	98.019775
rs13479277	7	46.313106	rs6377847	9	100.692014
rs6160140	7	53.312532	rs13480436	9	114.857586
rs3705155	7	55.55709	rs6302293	9	118.240473
rs6405142	7	55.615198	gnf09.117.044	9	119.540217
rs13479317	7	56.524353	gnf10.004.219	10	3.088178
rs3693876	7	56.589457	rs13480480	10	7.4464
gnf07.064.092	7	56.675124	rs13480493	10	9.914514
rs13479319	7	56.83062	rs6192001	10	12.394109
rs3657147	7	57.152088	rs13480506	10	14.854057
rs13479321	7	57.294626	rs3696055	10	17.883074
rs13479334	7	60.382457	rs13480525	10	18.959895
mCV25303361	7	60.653332	rs6410821	10	21.805932
rs13479338	7	61.105319	rs13480606	10	47.620358
rs3716002	7	61.171781	rs13480629	10	67.390746
rs13479425	7	86.603215	CEL-10_73933097	10	73.933097
rs13479427	7	87.305015	rs3165937	10	75.71362
rs13479506	7	112.15241	rs13480674	10	83.171156
rs3687061	7	115.134183	rs13480720	10	97.074107
gnf07.129.013	7	119.827503	rs3705990	10	99.186438
rs3663988	7	126.965988	mCV24206699	10	106.695924
rs3694208	8	14.730616	CEL-10_109935001	10	109.935001
rs13479627	8	18.663422	CEL-10_119602638	10	119.602638
rs13479741	8	46.240949	rs13480804	10	123.197828
mCV23056731	8	55.932578	rs13480818	10	126.541735
rs3726906	8	58.201849	rs13480839	11	4.125911
UT_8_61.137722	8	60.222067	rs13480847	11	6.162191
rs3712611	8	65.261034	rs13480859	11	8.373216
rs13479813	8	66.929486	rs13480869	11	10.898579
rs3690549	8	76.12653	mCV23851630	11	13.163316
rs6296891	8	79.15201	CEL-11_15345124	11	15.345124
rs8236770	8	82.939075	rs13480889	11	17.360319
rs13479880	8	86.040626	rs3678321	11	19.522724
rs13480026	8	123.079487	rs6367881	11	26.054403
rs4227456	8	124.053484	rs3657760	11	32.856509
rs3711756	9	34.437206	rs13480965	11	35.564301
rs3669224	9	37.029046	rs13480997	11	43.490965
rs13480151	9	40.150719	mCV23044839	11	65.492498
rs13480160	9	42.760021	UT_11_68.607315	11	68.648705
CEL-9_49033157	9	49.033157	gnf11.079.978	11	75.081947
gnf09.058.846	9	64.854283	rs13481119	11	79.159755
rs6174757	9	68.381485	rs13481123	11	81.870816
rs13480267	9	72.087091	gnf11.093.966	11	86.965398
rs13480277	9	74.403157	rs13481154	11	90.012989

rs3714299	11	92.73468	mCV22624058	13	58.311395
rs3710148	11	96.196554	CEL-13_60831741	13	60.831741
rs3695865	11	101.350115	rs6179438	13	68.057256
rs6384437	11	103.833223	rs13481878	13	70.156974
rs13481226	11	109.851386	rs3669221	13	80.367567
rs13481233	11	111.472921	rs13481918	13	81.984151
rs13481264	11	119.95554	rs3655061	13	86.241392
rs13481276	12	3.64518	rs4230027	13	97.200857
rs3699421	12	3.922406	rs3705092	13	100.020595
rs3655333	12	23.704034	rs13481992	13	102.442609
rs13481380	12	27.085682	rs13464858	13	105.049971
rs6187012	12	28.579503	rs13482011	13	107.432985
rs6223000	12	29.204179	rs4230094	13	110.039545
gnf12.033.545	12	30.14931	rs3657414	13	113.498433
rs6243157	12	32.296827	rs6340768	14	7.266803
rs3689063	12	50.059458	rs13482084	14	8.848582
rs13481465	12	50.509782	rs3719629	14	12.874743
rs3662939	12	54.082727	gnf14.019.954	14	22.731865
rs13481480	12	54.343362	rs13482122	14	27.054167
rs3674641	12	55.129109	rs6155573	14	29.837255
rs13481486	12	55.941001	rs3722090	14	32.565225
rs3721804	12	56.413174	mCV23384307	14	41.762771
rs13481488	12	56.527832	rs13482179	14	43.710903
rs13481499	12	59.639662	rs3140262	14	45.774656
rs3686891	12	60.735544	rs6392664	14	47.364134
rs4137680	12	61.533001	rs13482194	14	47.801247
rs3686378	12	63.477601	rs13482214	14	53.590138
rs13481522	12	66.97148	rs6161506	14	56.25048
rs13481531	12	69.626283	rs13482225	14	56.692261
mCV23169261	12	72.254494	rs6179045	14	57.017412
rs6318521	12	74.957568	CEL-14_65598536	14	65.598536
rs3696951	12	77.991846	rs3668373	14	66.081018
rs8259763	12	82.491932	rs6156908	14	66.473686
rs13481583	12	84.94215	gnf14.069.419	14	66.681374
rs13481589	12	86.779666	UT_14_66.802733	14	66.99281
rs3679514	12	92.576929	rs13482259	14	68.392545
rs3698001	12	95.092951	rs6298191	14	69.251728
gnf12.101.501	12	97.964429	CEL-14_71690454	14	71.690454
CEL-12_101776500	12	101.7765	rs3023412	14	72.111198
rs13481650	12	105.932708	CEL-14_72264521	14	72.264521
rs6198959	13	11.008611	rs6325141	14	73.875166
rs3721858	13	16.752051	rs13482273	14	74.295489
rs13481715	13	18.523821	rs13482301	14	81.862571
rs13481743	13	31.273115	rs3725470	14	82.281581
rs3727136	13	33.688045	gnf14.085.610	14	82.796068
gnf13.045.330	13	46.738859	rs6395984	14	82.944063
rs6209128	13	51.80716	rs6407863	14	83.212623
rs13481817	13	53.966943	rs13482306	14	83.42809
rs3700819	13	56.358945	rs3706761	14	83.865561

rs3655019	14	83.873573
rs6291434	14	83.88723
rs6176735	14	84.102001
rs6299927	14	84.309814
rs4139735	14	84.482998
rs13482309	14	84.549347
CEL-14_85152539	14	85.152539
rs13482311	14	85.28705
rs13482312	14	85.438583
rs3718262	14	85.692909
rs13482313	14	85.873792
rs13482314	14	86.050411
rs3708779	14	103.068
rs3683221	14	104.867978
rs3707842	14	111.967694
rs13482409	14	114.979966
rs13482416	14	117.011917
rs13482418	15	3.49896
rs3693019	15	7.948918
rs3714169	15	21.816884
rs13482490	15	26.813067
rs6210607	15	30.253148
rs6187312	15	31.266214
CEL-15_58115663	15	58.115663
rs3701449	15	60.283849
rs13482605	15	62.435922
rs13482618	15	66.126656
rs3697744	15	87.041436
rs3716673	15	92.579012
rs6285067	15	95.741617
rs13482734	15	99.184112
rs3690173	15	101.419686
mCV25142331	N/A	N/A
rs4166445	16	28.119031
rs4168890	16	31.10743
rs4170974	16	32.825259
rs4172915	16	35.504262
rs4174174	16	37.637201
rs4175353	16	38.902653
rs4197416	16	66.683459
rs3656592	16	87.019209
rs4217061	16	90.320114
rs4220668	16	95.392761
rs3164088	16	97.1111
rs3694629	17	6.436726
rs3702484	17	9.103503
mCV22941359	17	9.24339
rs3662575	17	9.388595
rs6309949	17	10.69913
rs3674166	17	11.214621
rs3674900	17	11.922826
gnf17.011.487	17	12.376655
rs3723317	17	12.406402
rs6270865	17	15.307109
rs3667748	17	15.457982
rs3721884	17	16.225206
rs4231344	17	22.269867
rs3724223	17	32.068525
UT_17_33.238924	17	32.099572
rs8242408	17	32.690515
mhcTNFa7	17	33.6972
rs3682923	17	34.343989
rs6298471	17	35.059374
rs13482974	17	36.98639
rs8254221	17	38.94185
mCV25197172	17	41.0809
rs8273969	17	52.02091
rs13483042	17	53.781849
mCV22888090	17	61.947366
rs3675634	17	69.670995
rs13483139	17	83.294259
rs3667051	17	88.400292
CEL-17_91401354	17	91.401354
rs13483179	17	93.216106
rs13483210	18	12.147024
rs13483217	18	14.478102
rs13483230	18	17.674697
rs3656185	18	19.941646
rs3691362	18	30.05309
rs3714096	18	36.136675
rs6302276	18	39.612385
rs13483319	18	41.351739
gnf18.043.155	18	45.21264
rs6334596	18	55.31767
rs13483378	18	57.789927
rs6364818	18	59.921604
rs6350869	18	61.07351
rs6346101	18	69.04019
rs13483433	18	72.937807
rs13483466	18	82.712912
rs13483472	18	84.261455
rs13483500	19	3.744813
rs3671671	19	4.208173
rs3713033	19	4.818261
rs13483505	19	4.836043
rs13483510	19	6.160915
rs13483511	19	6.331751

rs3023477	19	7.133376	rs13483834	X	67.880612
CEL-19_8529644	19	8.529644	CEL-X_68179178	X	68.179178
UT_19_10.709331	19	9.813271	CEL-X_68645226	X	68.645226
rs6163293	19	9.899424	rs13483838	X	68.74927
rs3700209	19	10.19721	CEL-X_71104123	X	71.104123
rs3688406	19	11.531705	CEL-X_71438949	X	71.438949
rs6237846	19	12.452484	rs13483849	X	71.752528
CEL-19_12595293	19	12.595293	CEL-X_72627341	X	72.627341
CEL-19_12911424	19	12.911424	CEL-X_72697823	X	72.697823
rs3692733	19	13.056443	CEL-X_73027245	X	73.027245
rs3694570	19	13.056716	gnfX.070.167	X	73.485959
rs3662712	19	15.964901	rs13483858	X	73.756646
rs3669192	19	16.124735	CEL-X_74073918	X	74.073918
rs3686467	19	16.159229	rs13483862	X	74.580193
rs8267310	19	16.991104	rs13483863	X	74.827617
rs13483555	19	19.177412	CEL-X_74985293	X	74.985293
gnf19.017.711	19	19.28395	CEL-X_75125049	X	75.125049
rs3720318	19	19.458954	rs13483864	X	75.392167
rs13483557	19	19.479228	rs13483877	X	78.499387
rs13483563	19	20.842526	rs13483803	X	81.225989
rs6392565	19	21.461474	gnfX.076.619	X	84.227028
rs3672759	19	21.707322	rs13483888	X	86.725106
rs3653630	19	21.729294	rs13483898	X	89.327593
rs13483571	19	23.62697	CEL-X_91222960	X	91.22296
rs13483577	19	25.63107	CEL-X_94143306	X	94.143306
rs3090325	19	26.007713	rs13483927	X	97.613903
rs13483669	19	51.559161	rs13483935	X	101.057496
rs6257938	19	52.744319	rs13483941	X	102.845364
CEL-X_44311522	X	44.311522	rs6221690	X	121.042456
rs6411410	X	44.405254	rs13484004	X	123.782683
rs13483753	X	44.656596	CEL-X_125736335	X	125.736335
rs13483756	X	45.475262	rs13484023	X	127.710219
rs13483757	X	45.612522	rs13484031	X	129.847872
rs13483771	X	51.03826	rs13484040	X	131.961674
CEL-X_51185805	X	51.185805	rs13484043	X	132.699243
rs13483777	X	52.741694	rs13484094	X	147.274623
rs13483778	X	53.039702	rs6365259	X	150.148872
rs13483831	X	67.285372	CEL-X_158112484	X	158.112484
