# Supplementary figures and images for: Quantitative trait loci for energy balance traits in an advanced intercross line derived from mice divergently selected for heat loss
Source: PeerJ. 2014 May 27;2:e392. doi: 10.7717/peerj.392 (PMC4045330; doi:10.7717/peerj.392)

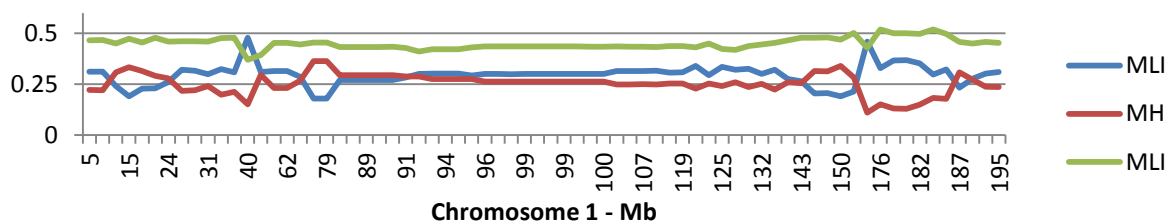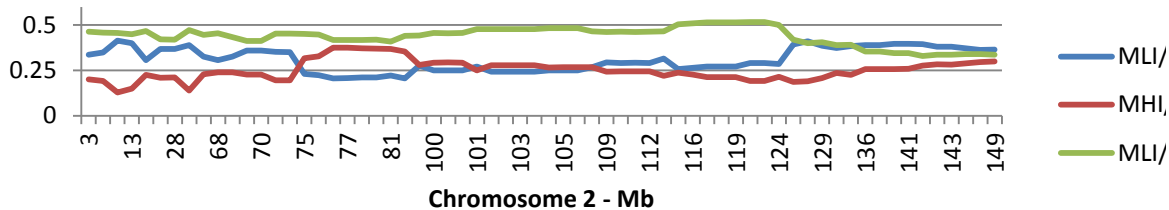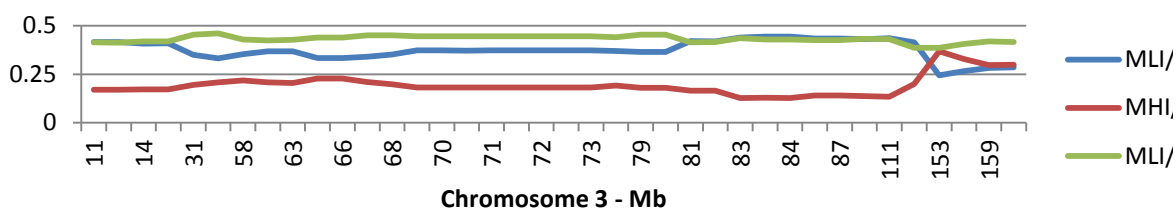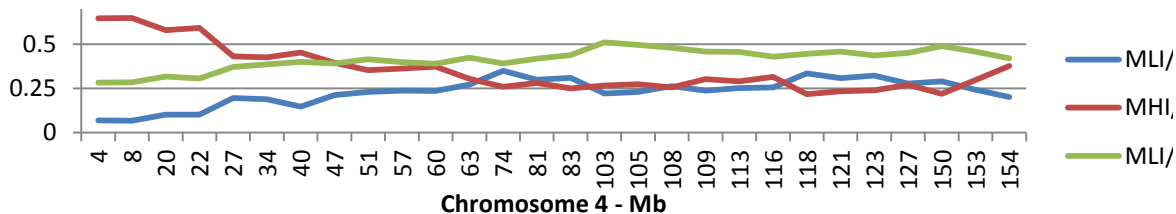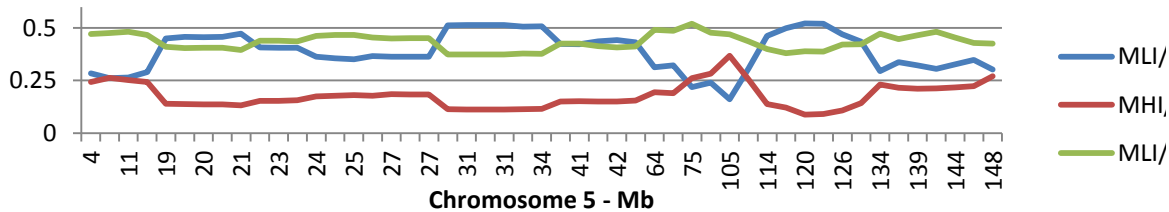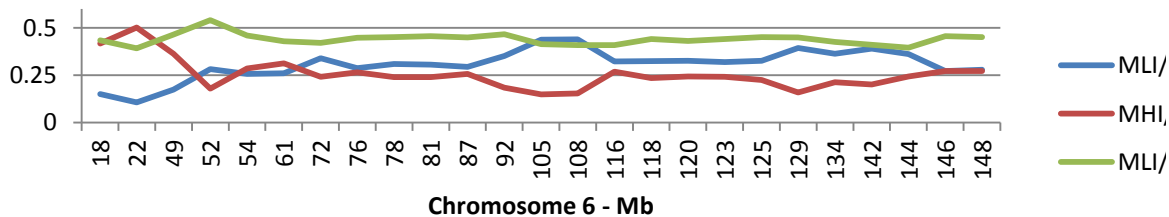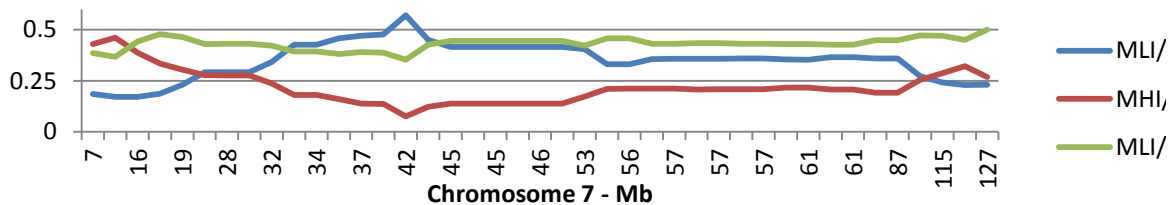

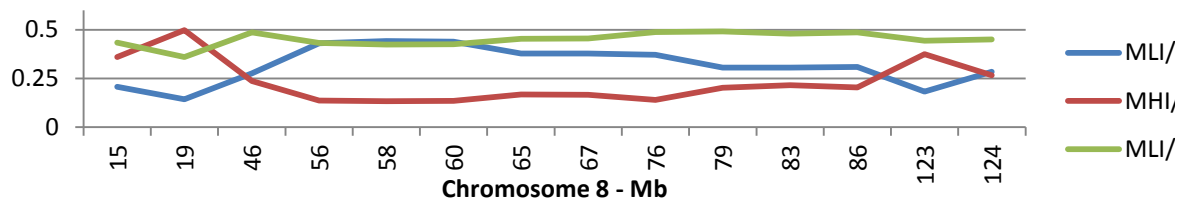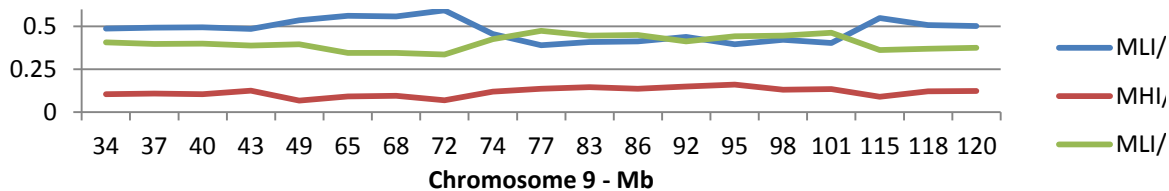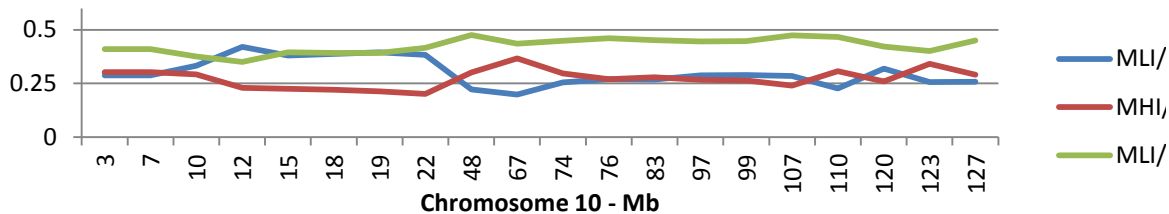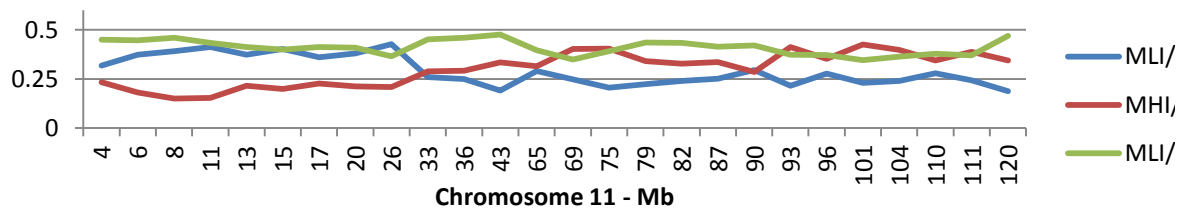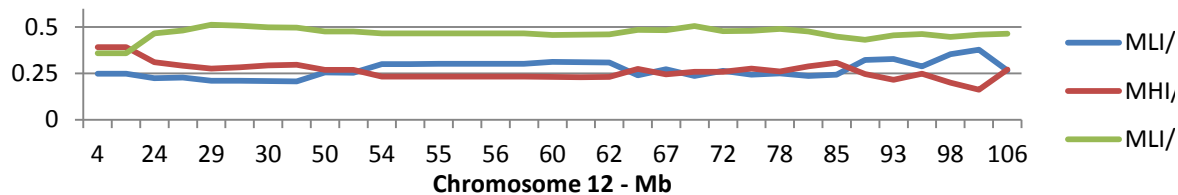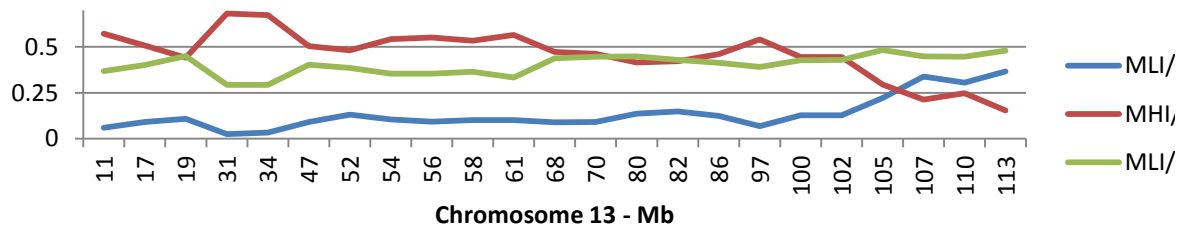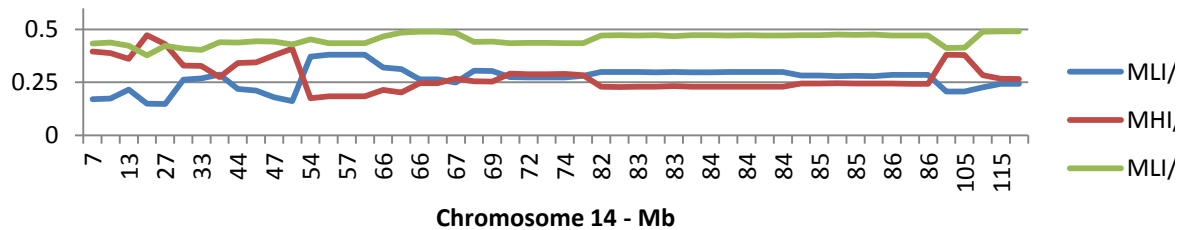

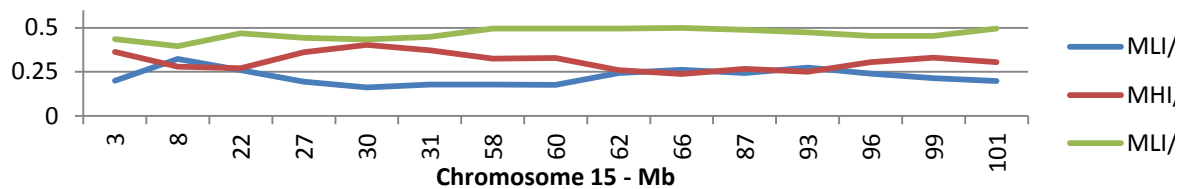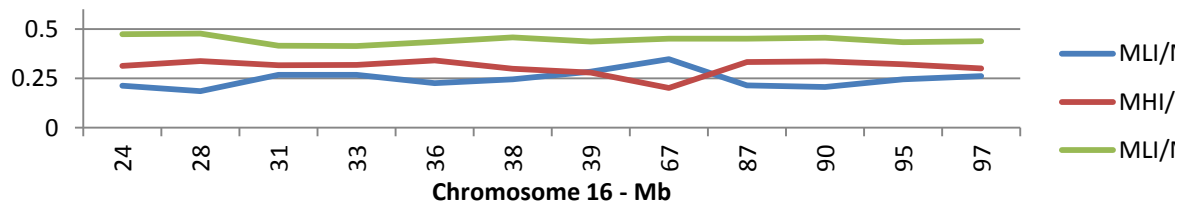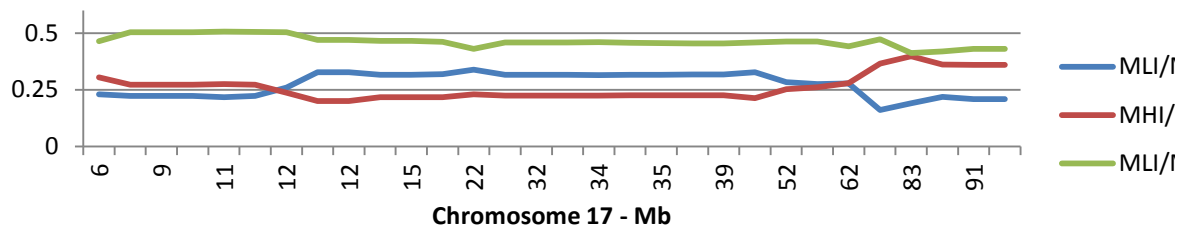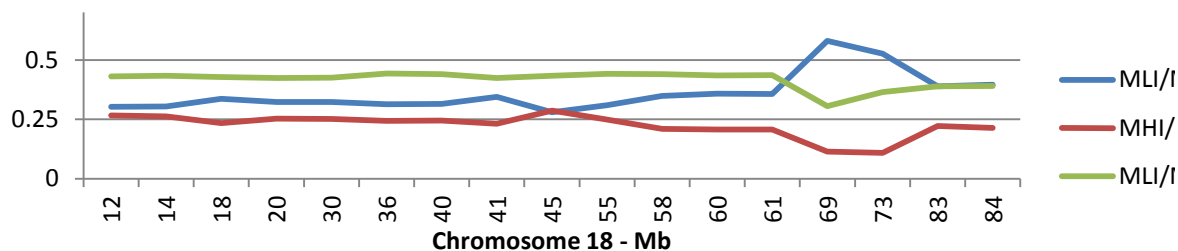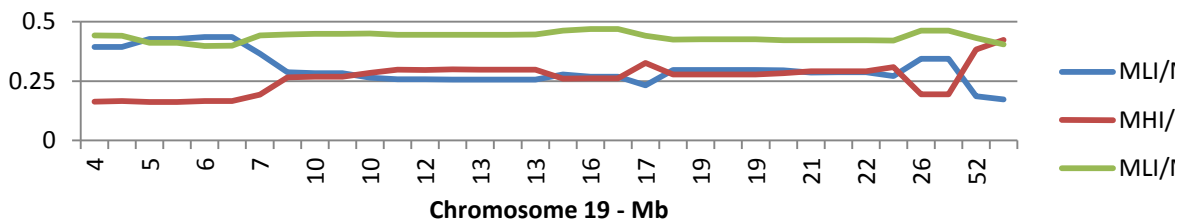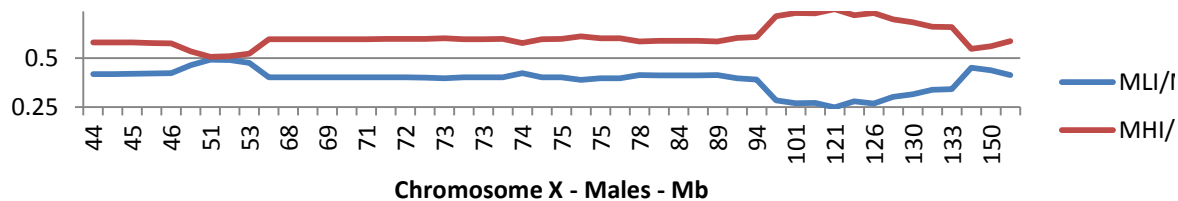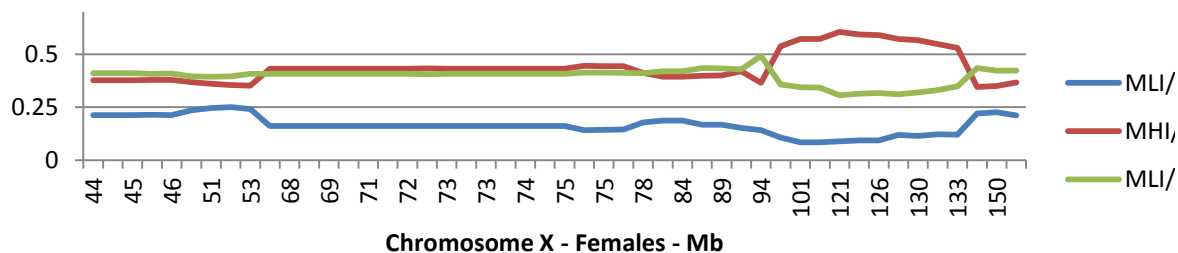

Supplement: Appendix S2 — Mb = megabases. [file peerj-02-392-s002.pdf]
