# Supplementary material for: Quantitative trait loci for energy balance traits in an advanced intercross line derived from mice divergently selected for heat loss
Source: PeerJ. 2014 May 27;2:e392. doi: 10.7717/peerj.392 (PMC4045330; doi:10.7717/peerj.392)
Supplement: Supplemental Information 3 [file peerj-02-392-s003.pdf]

APPENDIX 3. EXAMPLE OF QTLREL CODE

```
library(QTLRel)
setwd("C:/Active/Papers/QTL/POMPF11/Data/")

#Get data in to R
pedf10 = read.table("PEDIGREE.txt",header=TRUE,check.names=FALSE)
gdat = read.table("GENOTNEW.txt",header=TRUE,check.names=FALSE)
pdat = read.table("PHENOT.txt",header=TRUE,check.names=FALSE, na.str="NA")
gmap = read.table("MAP.txt",header=TRUE,check.names=FALSE)
str(pedf10)
str(gdat)
str(pdat)
str(gmap)


#Calculate identity coefficients
id = rownames(pdat)
id[1:5]
sum(!is.element(id,rownames(gdat)))
sum(!is.element(rownames(gdat),id))
sum(!is.element(id,pedf10$id))
idcf = cic(pedf10,ids=id,df=3,ask=TRUE,verbose=TRUE)

#Extract genetic matrices
gmf10 = genMatrix(idcf); names(gmf10)
dim(gmf10$AA)
gmf10$AA[1:3,1:5]


#Estimate variance components
idx = !is.na(pdat[,"6-12gain"])
pdatTmp = pdat[idx,]
gdatTmp = gdat[match(rownames(pdatTmp),rownames(gdat)),]
ii = match(rownames(pdatTmp),rownames(gmf10$AA))
vc = estVC(y = pdatTmp[,"6-12gain"],x = pdatTmp[,c("Sex", "Rep", "Parity")],
v = list(AA=gmf10$AA[ii,ii], DD = gmf10$DD[ii,ii], HH=NULL,AD=NULL,MH=NULL,
EE=diag(nrow(pdatTmp))))
vc$value
vc$par

#Impute genotypic probabilities
gdatTmpImputed = genoImpute(gdatTmp,gmap,gr=10,na.str=0)


# Haley-Knott scan
```

```
gdTmp = gdatTmpImputed
gdTmp[is.na(gdTmp)] = 0
unique(c(as.matrix(gdTmp)))
prDat = genoProb(gdat=gdTmp,gmap=gmap,step=3,gr=10)
lrtHK = scanOne(y=pdatTmp[,"6-12gain"], x = pdatTmp[,c("Sex", "Rep", "Parity")],
prd=prDat, vc=vc)
plot(lrtHK,main="6-12gain")


# Find locations of peak LRT scores
idx = match(colnames(gdatTmpImputed),gmap$snp)
Tmp<- data.frame(chr=lrtHK$chr,
                 dist=lrtHK$dist,
                 y=lrtHK$p/(2*log(10))) # convert to LOD
   Tmp$chr<- reorder(Tmp$chr)
   Tmp<- Tmp[order(Tmp$chr,Tmp$dist),] # order by chromosome and distance
lc<- lodci(Tmp,cv=3.7,lod=1.5,drop=1.5)
lc

lrtHK$dist[335]
lrtHK$p[335]/4.6052
lrtHK$dist[971]
lrtHK$p[971]/4.6052
lrtHK$dist[1455]
lrtHK$p[1455]/4.6052
lrtHK$dist[2188]
lrtHK$p[2188]/4.6052
lrtHK$dist[1534]
lrtHK$p[1534]/4.6052
lrtHK$dist[2156]
lrtHK$p[2156]/4.6052
lrtHK$dist[2187]
lrtHK$p[2187]/4.6052
lrtHK$dist[2190]
lrtHK$p[2190]/4.6052


# Estimate a and d effects with standard errors
dtfTmp<- data.frame(
  y=pdatTmp[,"6-12gain"],
  Sex=pdatTmp[,"Sex"],
  Rep=pdatTmp[,"Rep"],
 Parity=pdatTmp[,"Parity"],
   a1=prDat$pr[,1,lc$index[1]]-prDat$pr[,3,lc$index[1]],
  d1=prDat$pr[,2,lc$index[1]]
  )
```

```
est1<- gls(y~Sex+Rep+Parity+a1+d1,data=dtfTmp,vc=vc)
est1

# Estimate a and d effects with standard errors
dtfTmp<- data.frame(
  y=pdatTmp[,"6-12gain"],
  Sex=pdatTmp[,"Sex"],
  Rep=pdatTmp[,"Rep"],
  Parity=pdatTmp[,"Parity"],
   a1=prDat$pr[,1,lc$index[2]]-prDat$pr[,3,lc$index[2]],
  d1=prDat$pr[,2,lc$index[2]]
  )
est1<- gls(y~Sex+Rep+Parity+a1+d1,data=dtfTmp,vc=vc)
est1

# Estimate a and d effects with standard errors
dtfTmp<- data.frame(
  y=pdatTmp[,"6-12gain"],
  Sex=pdatTmp[,"Sex"],
  Rep=pdatTmp[,"Rep"],
  Parity=pdatTmp[,"Parity"],
    a1=prDat$pr[,1,lc$index[3]]-prDat$pr[,3,lc$index[3]],
  d1=prDat$pr[,2,lc$index[3]]
  )
est1<- gls(y~Sex+Rep+Parity+a1+d1,data=dtfTmp,vc=vc)
est1

# Estimate a and d effects with standard errors
dtfTmp<- data.frame(
  y=pdatTmp[,"6-12gain"],
  Sex=pdatTmp[,"Sex"],
  Rep=pdatTmp[,"Rep"],
  Parity=pdatTmp[,"Parity"],
    a1=prDat$pr[,1,lc$index[4]]-prDat$pr[,3,lc$index[4]],
  d1=prDat$pr[,2,lc$index[4]]
  )
est1<- gls(y~Sex+Rep+Parity+a1+d1,data=dtfTmp,vc=vc)
est1

# Estimate a and d effects with standard errors
dtfTmp<- data.frame(
  y=pdatTmp[,"6-12gain"],
  Sex=pdatTmp[,"Sex"],
  Rep=pdatTmp[,"Rep"],
  Parity=pdatTmp[,"Parity"],
  a1=prDat$pr[,1,lc$index[5]]-prDat$pr[,3,lc$index[5]],
```

```
  d1=prDat$pr[,2,lc$index[5]]
  )
est1<- gls(y~Sex+Rep+Parity+a1+d1,data=dtfTmp,vc=vc)
est1

# Estimate a and d effects with standard errors
dtfTmp<- data.frame(
  y=pdatTmp[,"6-12gain"],
  Sex=pdatTmp[,"Sex"],
  Rep=pdatTmp[,"Rep"],
  Parity=pdatTmp[,"Parity"],
    a1=prDat$pr[,1,lc$index[6]]-prDat$pr[,3,lc$index[6]],
  d1=prDat$pr[,2,lc$index[6]]
  )
est1<- gls(y~Sex+Rep+Parity+a1+d1,data=dtfTmp,vc=vc)
est1

# Estimate a and d effects with standard errors
dtfTmp<- data.frame(
  y=pdatTmp[,"6-12gain"],
  Sex=pdatTmp[,"Sex"],
  Rep=pdatTmp[,"Rep"],
  Parity=pdatTmp[,"Parity"],
    a1=prDat$pr[,1,lc$index[7]]-prDat$pr[,3,lc$index[7]],
  d1=prDat$pr[,2,lc$index[7]]
  )
est1<- gls(y~Sex+Rep+Parity+a1+d1,data=dtfTmp,vc=vc)
est1

# Estimate a and d effects with standard errors
dtfTmp<- data.frame(
  y=pdatTmp[,"6-12gain"],
  Sex=pdatTmp[,"Sex"],
  Rep=pdatTmp[,"Rep"],
  Parity=pdatTmp[,"Parity"],
    a1=prDat$pr[,1,lc$index[8]]-prDat$pr[,3,lc$index[8]],
  d1=prDat$pr[,2,lc$index[8]]
  )
est1<- gls(y~Sex+Rep+Parity+a1+d1,data=dtfTmp,vc=vc)
est1


# Calculate %variation generated by QTL
ii<- match(rownames(pdatTmp),rownames(gmf10$AA))
vc0<- estVC(y = pdatTmp[,"6-12gain"], v=list(AA=gmf10$AA[ii,ii],
   DD=gmf10$DD[ii,ii], HH=NULL,AD=NULL, MH=NULL,
```

```
   EE=diag(nrow(pdatTmp))))
nb<- length(vc0$par) - sum(vc0$nnl)
nr<- nrow(vc0$y)
cov<- matrix(0,nrow=nr,ncol=nr)
for(i in 1:vc0$nv)
   if(vc0$nnl[i]) cov<- cov + vc0$v[[i]]*vc0$par[nb+vc0$nn[i]]
tv<- mean(diag(cov)) # total variation
eff<- NULL # QTL effects
for(n in 1:length(lrtHK$par)){
   eff<- rbind(eff,lrtHK$par[[n]][c("a","d")])
}
eff<- data.frame(eff) # data frame!
qv<- qtlVar(eff,prDat$pr) # per QTL variation
qv[lc$index]/tv*100 # per QTL heritability


# Test for QTL by Sex interaction
lrtSEX<- scanOne(y=pdatTmp[,"6-12gain"], x = pdatTmp[,c("Rep", "Parity")],
    prd=prDat, intcovar=pdatTmp[,c("Sex")],vc=vc)
lrtHKTmp2 <- lrtHK
   lrtHKTmp2$p<- lrtSEX$p - lrtHK$p

#Put the index value at the peak position in parentheses below to get position and LOD

lrtHKTmp2$p[335]/4.6052
lrtHKTmp2$p[335]/4.6052
lrtHKTmp2$p[1455]/4.6052
lrtHKTmp2$p[2188]/4.6052


# Permutation testing -HK approach
nn<- nrow(gdatTmp) # sample size
ntimes<- 1000 # number of simulations
cvMtrHK = NULL
for(n in 1:ntimes){
idx = sample(1:nn,replace=FALSE)
prdTmp = prDat
prdTmp$pr = prdTmp$pr[idx,,]
tmp = scanOne(y=pdatTmp[,"6-12gain"], x = pdatTmp[,c("Sex", "Rep", "Parity")],
prd=prdTmp, vc=vc)
cvMtrHK = rbind(cvMtrHK,tmp$p)
cat(n,"/",ntimes,"\r")
}
00/1000
```

```
#Find thresholds
# 0.05 thresholds
quantile(apply(cvMtrHK,1,max),0.95)/(2*log(10))
# 0.10 thresholds
quantile(apply(cvMtrHK,1,max),0.90)/(2*log(10))
#chromosome 1
quantile(apply(cvMtrHK[,1:181],1,max),0.95)/(2*log(10))
#chromosome 2
quantile(apply(cvMtrHK[,182:314],1,max),0.95)/(2*log(10))
#chromosome 3
quantile(apply(cvMtrHK[,315:458],1,max),0.95)/(2*log(10))
#chromosome 4
quantile(apply(cvMtrHK[,459:596],1,max),0.95)/(2*log(10))
#chromosome 5
quantile(apply(cvMtrHK[,597:746],1,max),0.95)/(2*log(10))
#chromosome 6
quantile(apply(cvMtrHK[,747:868],1,max),0.95)/(2*log(10))
#chromosome 7
quantile(apply(cvMtrHK[,869:1001],1,max),0.95)/(2*log(10))
#chromosome 8
quantile(apply(cvMtrHK[,1002:1061],1,max),0.95)/(2*log(10))
#chromosome 9
quantile(apply(cvMtrHK[,1062:1157],1,max),0.95)/(2*log(10))
#chromosome 10
quantile(apply(cvMtrHK[,1158:1268],1,max),0.95)/(2*log(10))
#chromosome 11
quantile(apply(cvMtrHK[,1269:1394],1,max),0.95)/(2*log(10))
#chromosome 12
quantile(apply(cvMtrHK[,1395:1494],1,max),0.95)/(2*log(10))
#chromosome 13
quantile(apply(cvMtrHK[,1495:1587],1,max),0.95)/(2*log(10))
#chromosome 14
quantile(apply(cvMtrHK[,1588:1715],1,max),0.95)/(2*log(10))
#chromosome 15
quantile(apply(cvMtrHK[,1716:1812],1,max),0.95)/(2*log(10))
#chromosome 16
quantile(apply(cvMtrHK[,1813:1877],1,max),0.95)/(2*log(10))
#chromosome 17
quantile(apply(cvMtrHK[,1878:1977],1,max),0.95)/(2*log(10))
#chromosome 18
quantile(apply(cvMtrHK[,1978:2059],1,max),0.95)/(2*log(10))
#chromosome 19
quantile(apply(cvMtrHK[,2060:2140],1,max),0.95)/(2*log(10))
#chromosome X
```

```
quantile(apply(cvMtrHK[,2142:2211],1,max),0.95)/(2*log(10))
```
